# Metal‐Organic Framework Functionalized Bioceramic Scaffolds with Antioxidative Activity for Enhanced Osteochondral Regeneration

**DOI:** 10.1002/advs.202206875

**Published:** 2023-02-24

**Authors:** Chaoqin Shu, Chen Qin, Lei Chen, Yufeng Wang, Zhe Shi, Jiangming Yu, Jimin Huang, Chaoqian Zhao, Zhiguang Huan, Chengtie Wu, Min Zhu, Yufang Zhu

**Affiliations:** ^1^ State Key Laboratory of High Performance Ceramics and Superfine Microstructure Shanghai Institute of Ceramics Chinese Academy of Sciences Shanghai 200050 P. R. China; ^2^ Department of Orthopaedics Tongren Hospital Shanghai Jiaotong University Shanghai 200336 P. R. China; ^3^ Center of Materials Science and Optoelectronics Engineering University of Chinese Academy of Sciences Beijing 100049 P. R. China; ^4^ School of Materials and Chemistry University of Shanghai for Science and Technology Shanghai 200093 P. R. China

**Keywords:** antioxidative stress, bioceramic scaffolds, metal‐organic frameworks, osteochondral regeneration

## Abstract

Osteoarthritis (OA) is a degenerative disease that often causes cartilage lesions and even osteochondral damage. Osteochondral defects induced by OA are accompanied by an inflammatory arthrosis microenvironment with overproduced reactive oxygen species (ROS), resulting in the exacerbation of defects and difficulty regenerating osteochondral tissues. Therefore, it is urgently needed to develop osteochondral scaffolds that can not only promote the integrated regeneration of cartilage and subchondral bone, but also possess ROS‐scavenging ability to protect tissues from oxidative stress. Herein, zinc‐cobalt bimetallic organic framework (Zn/Co‐MOF) functionalized bioceramic scaffolds are designed for repairing osteochondral defects under OA environment. By functionalizing Zn/Co‐MOF on the 3D‐printed beta‐tricalcium phosphate (*β*‐TCP) scaffolds, the Zn/Co‐MOF functionalized β‐TCP (MOF‐TCP) scaffolds with broad‐spectrum ROS‐scavenging ability are successfully developed. Benefiting from its catalytic active sites and degradation products, Zn/Co‐MOF endows the scaffolds with excellent antioxidative and anti‐inflammatory properties to protect cells from ROS invasion, as well as dual‐bioactivities of simultaneously inducing osteogenic and chondrogenic differentiation in vitro. Furthermore, in vivo results confirm that MOF‐TCP scaffolds accelerate the integrated regeneration of cartilage and subchondral bone in severe osteochondral defects. This study offers a promising strategy for treating defects induced by OA as well as other inflammatory diseases.

## Graphical abstract

Zinc‐cobalt bimetallic organic framework (Zn/Co‐MOF) functionalized bioceramic scaffolds are developed by functionalizing Zn/Co‐MOF on the 3D‐printed beta‐tricalcium phosphate (*β*‐TCP) scaffolds. The Zn/Co‐MOF functionalized β‐TCP (MOF‐TCP) scaffolds possess the ability to protect cells from reactive oxygen species invasion, the immunomodulatory activity, as well as the dual bioactivity of simultaneously promoting osteogenesis and chondrogenesis, exhibiting great potential for treating osteochondral defects.

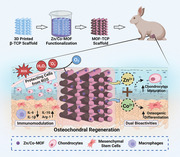

## 1 Introduction

Osteoarthritis (OA) is a prevalent degenerative disease causes joint damage. The patients who suffer OA are often faced with defective loss of cartilage and pathological injury of the underlying bone, leading to osteochondral defects.^[^ ^1^ ^]^ Studies have found that the progression and pathogenesis of OA are highly associated with the overproduction of reactive oxygen species (ROS) in arthrosis microenvironment.^[^ ^2^ ^]^ Oxidative stress induced by high levels of ROS will stimulate apoptosis of chondrocytes by destroying their mitochondrial deoxyribonucleic acid (DNA).^[^ ^3^ ^]^ Moreover, the overproduced ROS can also induce osteochondral degeneration by inhibiting the extracellular matrix (ECM) formation and facilitating ECM catabolism, and thereby exerting adverse impact on the regeneration of new tissues.^[^ ^4^ ^]^ Although some studies have developed kinds of osteochondral scaffolds for integrally repairing the cartilage and subchondral bone, OA microenvironment with excessive ROS may negatively affect the intrinsic properties of implanted scaffolds and hinder the formation of functional new tissues.^[^ ^5^ ^]^ Therefore, due to the complex component of osteochondral tissues and poor intraarticular environment with oxidative stress, the integrated regeneration of osteochondral tissues under OA environment is an urgent problem to be solved.

Bioceramic scaffolds were widely studied in bone tissue engineering field. Kinds of 3D‐printed bioceramic scaffolds were developed with dual bioactivities of repairing both subchondral bone and cartilage.^[^ ^6^ ^]^ Although these scaffolds possessed good mechanical properties and bioactivities, their performance under the OA environment with excessive ROS was largely ignored. Beta‐tricalcium phosphate (*β*‐TCP) is a typical bioceramic that has been extensively used as bone grafts.^[^ ^7^ ^]^ As previously reported, the degradation products of *β*‐TCP scaffolds, including calcium ion and phosphate ions, can participate in bone formation process.^[^ ^8^ ^]^ Moreover, by incorporating some specific elements, *β*‐TCP scaffolds with multifunction can be developed. For example, by introducing manganese into *β*‐TCP, the Mn‐TCP bioceramics were prepared to promote the osteoporotic bone regeneration with function of ROS scavenging.^[^ ^9^ ^]^ Besides, bone scaffolds combined magnesium with *β*‐TCP exhibited immunomodulatory properties and could adjust the immune microenvironment to be benefit for osteogenesis.^[^ ^10^ ^]^ Consequently, *β*‐TCP scaffolds with satisfactory mechanical properties and bioactivities can be an ideal matrix to be functionalized as osteochondral scaffolds with antioxidative ability.

Metal‐organic framework (MOF) is a porous hybrid material coordinated by metal ions/clusters and organic ligands.^[^ ^11^ ^]^ MOFs with broad‐spectrum ROS‐scavenging ability can be easily fabricated by integrating specific metal cations/clusters with organic ligands to construct catalytic active sites. Compared to natural enzymes, MOFs possess advantages of outstanding stability, biocompatibility, and biodegradability under physiological conditions.^[^ ^12^ ^]^ More importantly, with MOFs degradation, kinds of metal‐ions with bioactivities can release. Studies have found that zinc‐cobalt bimetallic MOF (Zn/Co‐MOF) with Zn and Co as their central metal cations exhibited excellent ROS catalytic activities similar to peroxidase (POD), catalase (CAT), and superoxide dismutase (SOD).^[^ ^13^ ^]^ Notably, Zn is an essential element with antioxidative and anti‐inflammatory properties by regulating free radicals formation in human body.^[^ ^14^ ^]^ Previous studies have found that Zn could be beneficial for cartilage formation due to its insulin‐mimetic properties.^[^ ^15^ ^]^ Besides, Co^2+^ was verified to have positive effects on promoting angiogenesis and osteogenesis.^[^ ^16^ ^]^ Therefore, modifying *β*‐TCP scaffolds with Zn/Co‐MOF is promising to construct scaffolds with capabilities of scavenging ROS and promoting regeneration of both cartilage and bone, which is suitable for treating osteochondral defects induced by OA.

**Scheme 1:**
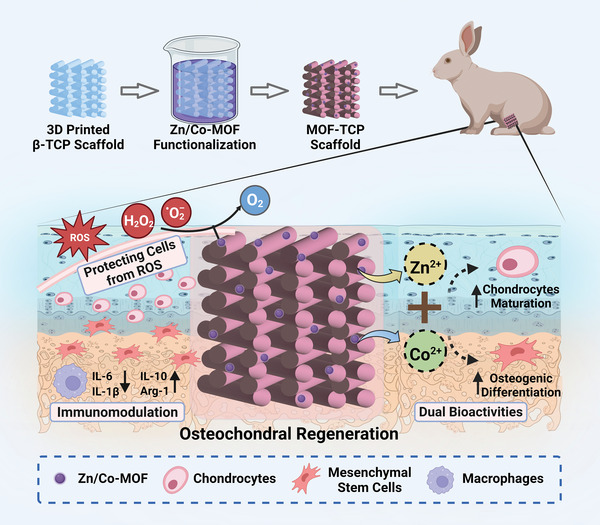
Illustration diagrams of metal‐organic framework tricalcium phosphate (MOF‐TCP) scaffolds in treating osteochondral defects caused by osteoarthritis (OA). With zinc‐cobalt bimetallic organic framework (Zn/Co‐MOF) functionalization, the scaffolds could scavenge excessive reactive oxygen species (ROS) and build an anti‐inflammatory immune microenvironment to protect cells. Meanwhile, MOF‐TCP scaffolds exhibited dual bioactivities of stimulating the cells toward specific differentiation by releasing multiple bioactive ions. Briefly, the MOF‐TCP scaffolds, which possessed ROS‐scavenging abilities, ion release properties, and immunomodulatory activities, exhibited ideal function of promoting osteochondral regeneration.

Herein, by functionalizing *β*‐TCP scaffolds with Zn/Co‐MOF, we successfully developed osteochondral scaffolds with ROS‐scavenging capability for treating OA defects. As shown in **Scheme** **1**, the Zn/Co‐MOF functionalized *β*‐TCP scaffolds (MOF‐TCP) were firstly prepared via in situ deposition of Zn/Co‐MOF on 3D‐printed *β*‐TCP scaffolds. By adjusting the concentration of Zn/Co‐MOF reaction solution, their antioxidative ability of scavenging multiple ROS and cytocompatibility could be easily modulated. Then, the in vitro bioactivities of MOF‐TCP scaffolds under oxidative stress were testified. On the one hand, Zn/Co‐MOF allowed MOF‐TCP scaffolds to protect both chondrocytes and mesenchymal stem cells from oxidative injury by scavenging ROS. On the other hand, multiple bioactive ions released by the scaffolds could stimulate the cells toward specific differentiation. Moreover, the expression of inflammatory factors was inhibited as the ROS levels decreasing, indicating that MOF‐TCP scaffolds could mediate an anti‐inflammatory microenvironment under OA conditions, which in turn favor the ROS elimination and new tissue formation. At last, the positive effects of MOF‐TCP scaffolds on integrally regenerating osteochondral tissues were demonstrated in the rabbit osteochondral defect models. Taken together, the Zn/Co‐MOF functionalized *β*‐TCP scaffolds with antioxidative ability and dual‐bioactivities offered a promising strategy for treatment of osteochondral defects caused by OA.

## 2 Results and Discussion

### 2.1 Preparation of Zn/Co‐MOF Functionalized *β*‐TCP Scaffolds (MOF‐TCP) with Broad‐Spectrum Antioxidative Abilities

**Figure 1:**
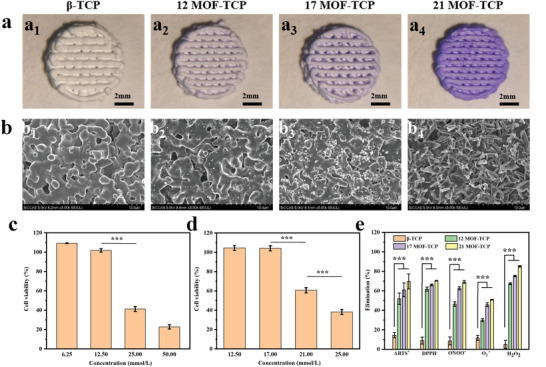
Characterization of the 3D‐printed beta‐tricalcium phosphate (*β*‐TCP) scaffolds functionalized with different amount of zinc‐cobalt bimetallic organic framework (Zn/Co‐MOF). a) Digital photographs of scaffolds with different levels of Zn/Co‐MOF functionalization. b) Scanning electron microscope (SEM) images of the surfaces of scaffolds. c) Cell viability of MOF‐TCP scaffolds prepared by Zn/Co‐MOF reaction solution with concentrations of 6.25, 12.5, 25, 50 × 10^−3^ m (*n* = 4). d) Cell viability of MOF‐TCP scaffolds prepared by Zn/Co‐MOF reaction solution with concentrations of 12, 17, 21, 25 × 10^−3^ m (*n* = 4). e) Multiple reactive oxygen species (ROS) elimination ratios of the scaffolds (*n* = 5). *p < 0.05, **p < 0.01, ***p < 0.001.

To fabricate *β*‐TCP scaffolds functionalized with Zn/Co‐MOF, pure *β*‐TCP scaffolds were prepared by 3D printing and then Zn/Co‐MOF was in situ deposited on their surface through soaking the *β*‐TCP scaffolds in the Zn/Co‐MOF reaction solution. The Zn/Co‐MOF reaction solution was obtained by adding Zn(NO_3_)_2_·6H_2_O, Co(NO_3_)_2_·6H_2_O and 2‐methylimidazole (2‐MIM) into deionized water. By adjusting the concentrations of Zn/Co‐MOF reaction solution, the 3D scaffolds with different amount of Zn/Co‐MOF were prepared. According to digital photos (**Figure** **1**a1–a4), the scaffolds were 7.5 ± 0.5 mm in diameter and 1.9 ± 0.2 mm in height. Among them, *β*‐TCP represented the scaffolds without Zn/Co‐MOF functionalization, 12MOF‐TCP, 17MOF‐TCP, and 21MOF‐TCP represented the scaffolds reacted in the Zn/Co‐MOF reaction solution with the ion concentrations of 12 × 10^−3^, 17 × 10^−3^ , and 21 × 10^−3^ m, respectively. With the concentration increasing, color of the MOF‐TCP scaffolds gradually changed from white to intense purple. In order to investigate the interior structure of the scaffolds, 17MOF‐TCP scaffolds with diameter of 7.5 ± 0.5 mm and height of 10 ± 0.5 mm were prepared and scanned by microcomputed tomography (Micro‐CT). As shown in Figure S1 (Supporting Information), the cross‐section view and vertical section view of the scaffolds indicated that the scaffolds displayed three‐dimensional connected macropore structure. Based on the 3D reconstructed analysis, the total porosity of the scaffolds was 30.1 ± 0.9% (*n* = 4). Observed by scanning electron microscope (SEM), the size and number of nanoparticles formed on the surface of *β*‐TCP scaffolds increased with the increase of Zn/Co‐MOF solution concentration (Figure 1b1–b4). When the solution concentration increased to 21 × 10^−3^ m, the surfaces of scaffolds were fully covered by some flaky substance. Subsequently, X‐ray diffraction (XRD) analysis was conducted to identify the composition of these substance generated on scaffolds. The XRD patterns (Figure S2a, Supporting Information) showed that the characteristic peaks of Zn/Co‐MOF at 2*θ* = 10.8, 12.6, 14.8, and 18.0 could be observed in the three groups of scaffolds with Zn/Co‐MOF functionalization. The mechanical property analysis showed that the compressive strength of MOF‐TCP scaffolds was slightly reduced after being functionalized with Zn/Co‐MOF (Figure S2b, Supporting Information), which might be attributed to the slight degradation of the scaffolds during the preparation process. The *β*‐TCP scaffolds could dissolve in the reaction solution under the continuous stirring. And, with the concentration of Zn/Co‐MOF reaction solution increasing, the solution appeared to be more acidic and caused more degradation of the *β*‐TCP scaffolds, leading to the reduction of compressive strength. Moreover, the intensity of these characteristic peaks gradually increased as the concentration of reaction solutions increasing. Energy dispersive spectroscopy (EDS) analysis was further conducted to evaluate the surface composition and elemental distribution of the 17MOF‐TCP scaffolds. As shown in Figure S3 (Supporting Information), Ca, P, C, O, N, Zn, and Co element uniformly distributed on the scaffolds. It could be inferred that Ca, P, and O were the constituent elements of *β*‐TCP matrix. Zn, Co, N, and C were the constituent elements of the generated Zn/Co‐MOF. Accordingly, these results proved that Zn/Co‐MOF was successfully in situ deposited on the surface of *β*‐TCP scaffolds. Furthermore, more Zn/Co‐MOF nanoparticles were produced on the surface of *β*‐TCP scaffolds as the concentration of reaction solution increased.

Besides, the release profiles of Ca^2+^, (PO_4_)^3−^, Zn^2+^, and Co^2+^ of MOF‐TCP scaffolds were evaluated by soaking them in the Tris‐HCl solution for 14 days. Figure S4 (Supporting Information) shows that MOF‐TCP scaffolds could gradually release Ca^2+^, (PO_4_)^3−^, Zn^2+^, and Co^2+^. More Zn^2+^ and Co^2+^ were released with the increase of Zn/Co‐MOF reaction concentration. These results indicated that both Zn/Co‐MOF and *β*‐TCP matrix of the scaffolds could continuously degrade and release bioactive ions. Interestingly, the three groups of MOF‐TCP scaffolds released fewer Ca^2+^ and (PO_4_)^3−^ ions than *β*‐TCP scaffolds. A reasonable explanation is that the surface functionalization of Zn/Co‐MOF weakened the interaction between the *β*‐TCP matrix and the Tris(hydroxymethyl)aminomethane (Tris‐HCl) solution.

Furthermore, in order to figure out the relationship between the cytocompatibility of MOF‐TCP scaffolds and concentration of Zn/Co‐MOF reaction solution, the MOF‐TCP scaffolds prepared with different concentration of Zn/Co‐MOF reaction solution (6.25 × 10^−3^–50 × 10^−3^ m) were seeded with rabbit bone marrow mesenchymal stem cells (rBMSCs). As shown in Figure 1c, when the concentration of the Zn/Co‐MOF reaction solution was higher than 25 × 10^−3^ m, the viability of rBMSCs was extremely poor. Meanwhile, when the concentration was lower than 12.5 × 10^−3^ m, the cell survival rate was close to 100%. Therefore, the concentration range of 12.5–25 × 10^−3^ m was subdivided for further investigation. As shown in Figure 1d, when the concentration of reaction solution reaches 21 × 10^−3^ m, the cell viability decreased to about 60%. Therefore, the MOF‐TCP scaffolds treated by Zn/Co‐MOF reaction solution with the concentrations of 12 × 10^−3^–21 × 10^−3^ m were suitable for cell survival.

Next, the broad‐spectrum antioxidative activities of the MOF‐TCP scaffolds were tested. As shown in Figures S5 and S6a (Supporting Information), MOF‐TCP scaffolds exhibited enhanced ability to scavenge 2,2ʹ‐azino‐bis(3‐ethylbenzothiazoline‐6‐sulphonic acid) (ABTS) free radicals with Zn/Co‐MOF increasing. Furthermore, their effects on scavenging diphenylpicrylhydrazyl (DPPH) free radicals further confirmed that the increased content of Zn/Co‐MOF enhanced the antioxidative activity of MOF‐TCP scaffolds (Figure S6b, Supporting Information). Subsequently, to evaluate the ability of scavenging nitroxide free radicals, MOF‐TCP scaffolds were added into the ONOO^−^ solution. UV–Vis analysis (Figure S6c, Supporting Information) confirmed that MOF‐TCP scaffolds showed obvious elimination effects on ONOO^−^. Similarly, the ONOO^−^ scavenging effects were positively correlated with the content of Zn/Co‐MOF. What's more, the SOD enzymes‐like activity of MOF‐TCP scaffolds was investigated by using a WST‐8 kit (Figure S6d, Supporting Information). The results showed that MOF‐TCP scaffolds could obviously scavenge O_2_^•−^ and exhibit excellent SOD activity compared to *β*‐TCP scaffolds. In addition, the CAT enzymes‐like activity of MOF‐TCP scaffolds was further demonstrated by reacting with hydrogen peroxide (H_2_O_2_). As shown in Figure S6e,f (Supporting Information), three groups of MOF‐TCP scaffolds could make the H_2_O_2_ decrease and generate lots of oxygen. The ROS elimination ratios of these four groups of scaffolds were summarized in Figure 1e. It could be seen that MOF‐TCP scaffolds could effectively scavenge multiple ROS, and their activity of catalyzing free radicals could be effectively regulated by adjusting the concentration of Zn/Co‐MOF reaction solution. On the contrary, *β*‐TCP scaffolds showed no catalytic activity on scavenging ROS and only removed a small amount of ROS by physical adsorption.

Briefly, MOF‐TCP scaffolds with moderate amount of Zn/Co‐MOF possessed satisfactory cell compatibility, ion release properties, and broad‐spectrum ROS‐scavenging ability. It can be inferred that MOF‐TCP scaffolds could scavenge varied ROS owing to the catalytic active sites constructed by Co and 2‐Methylimidazole (2‐MIM) in Zn/Co‐MOF. Previous study has found that Co nanoparticles possessed excellent antioxidative ability with dose‐dependent manner.^[^ ^17^ ^]^ In addition, Co single‐atom was found to possess enzymatic activities similar to SOD and CAT and exhibited efficient antioxidative abilities against O_2_^•‐^, ^•^OH, and H_2_O_2_.^[^ ^18^ ^]^ Therefore, with the increase of Zn/Co‐MOF, more catalytic active sites would exist and thus the scaffolds showed enhanced ROS scavenging activities. However, with the increase of Zn/Co‐MOF, the cell viability would be adversely affected. Encouragingly, by adjusting the concentration of Zn/Co‐MOF reaction solution, the ROS‐scavenging capacity and cell compatibility of the scaffolds could be easily optimized.

### 2.2 Promoting Effect of MOF‐TCP Scaffolds on the Proliferation, Adhesion, and Migration of rBMSCs and Chondrocytes under Oxidative Stress

**Figure 2:**
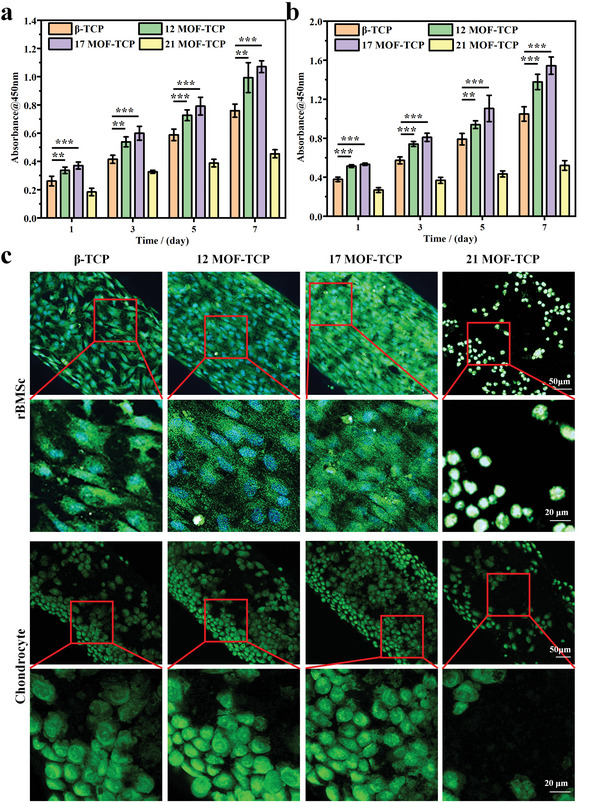
The promoting effects of MOF‐TCP scaffolds on cell proliferation and adhesion under oxidative stress. Proliferation of a) rabbit bone marrow mesenchymal stem cells (rBMSCs) and b) chondrocytes cultured on the scaffolds with hydrogen peroxide (H_2_O_2_) stimulation (*n* = 6). c) Morphology of rBMSCs and chondrocytes cultured on the scaffolds with H_2_O_2_ stimulation. *p < 0.05, **p < 0.01, ***p < 0.001.

To investigate the bioactivity of MOF‐TCP scaffolds under oxidative stress, rBMSCs and chondrocytes were cultured on the scaffolds with H_2_O_2_ adding in the culture medium. Shown in **Figure** **2**a,b, the proliferation of two types of cells was significantly promoted by 12MOF‐TCP and 17MOF‐TCP scaffolds. Meanwhile, the cells seeded on 21MOF‐TCP scaffolds could hardly proliferate and their viability was much lower than that of other groups. Besides, observed by confocal laser scanning microscope (CLSM), rBMSCs and chondrocytes adhered on 12MOF‐TCP and 17MOF‐TCP scaffolds were denser and showed more widespread cytoskeleton (Figure 2c). By analyzing the cells numbers in the magnified CLSM images (Figure S7a,b, Supporting Information), it could be found that more rBMSCs and chondrocytes were adhered to the 12MOF‐TCP and 17MOF‐TCP scaffolds compared with the *β*‐TCP scaffolds. In addition, the effects of MOF‐TCP scaffolds on the migration activities of rBMSCs and chondrocytes were investigated by the scratch test. Due to the cell activity was quite low in 21MOF‐TCP group, the migration activities of another three groups were investigated. Figure S7 (Supporting Information) shows that 12MOF‐TCP and 17MOF‐TCP scaffolds could significantly promote the migration of both rBMSCs and chondrocytes. These results demonstrated that MOF‐TCP scaffolds with suitable content of Zn/Co‐MOF could improve the proliferation, adhesion, and migration of both rBMSCs and chondrocytes under oxidative conditions.

To investigate the mechanism of MOF‐TCP scaffolds promoting cell proliferation and adhesion under oxidative condition, rBMSCs and chondrocytes were further cultured on four groups of scaffolds under normal conditions. Shown in Figure S6 (Supporting Information), the proliferation rates of both rBMSCs and chondrocytes seeded on 12MOF‐TCP and 17MOF‐TCP scaffolds were equivalent to that of *β*‐TCP scaffolds. Meanwhile, according to the CLSM images, rBMSCs and chondrocytes seeded on the 12MOF‐TCP and 17MOF‐TCP scaffolds showed similar spread cytoskeletons to the *β*‐TCP scaffolds. In addition, similar to the results under oxidative conditions, 21MOF‐TCP scaffolds apparently inhibited the proliferation and adhesion of these two cells.

Taken together, these results indicated that *β*‐TCP, 12MOF‐TCP, and 17MOF‐TCP scaffolds could well support the proliferation and adhesion of rBMSCs and chondrocytes under normal culture conditions. However, by comparing the results in Figure 2a,b and Figure S6a,b (Supporting Information), we found that the absorbance of *β*‐TCP group under oxidative condition was obviously lower than that under normal condition. Meanwhile, the absorbance of 12MOF‐TCP and 17MOF‐TCP groups showed no much difference between the two conditions. Hence, it could be inferred that the cells cultured with *β*‐TCP scaffolds which lacked ROS‐scavenging activity were hurted by H_2_O_2_. On the contrary, 12MOF‐TCP and 17MOF‐TCP scaffolds protected cells from oxidative stress by scavenging H_2_O_2_ in the culture medium, thus improving cell proliferation and adhesion activities. In addition, 21MOF‐TCP scaffolds inhibited proliferation and adhesion of the cells under both conditions. This might be ascribed to the high levels of Zn/Co‐MOF releasing too many degradation products such as Co^2+^ and Zn^2+^, which may cause cell injuries.

### 2.3 Promoting Effect of MOF‐TCP Scaffolds on the Specific Differentiation of rBMSCs and Chondrocytes

**Figure 3:**
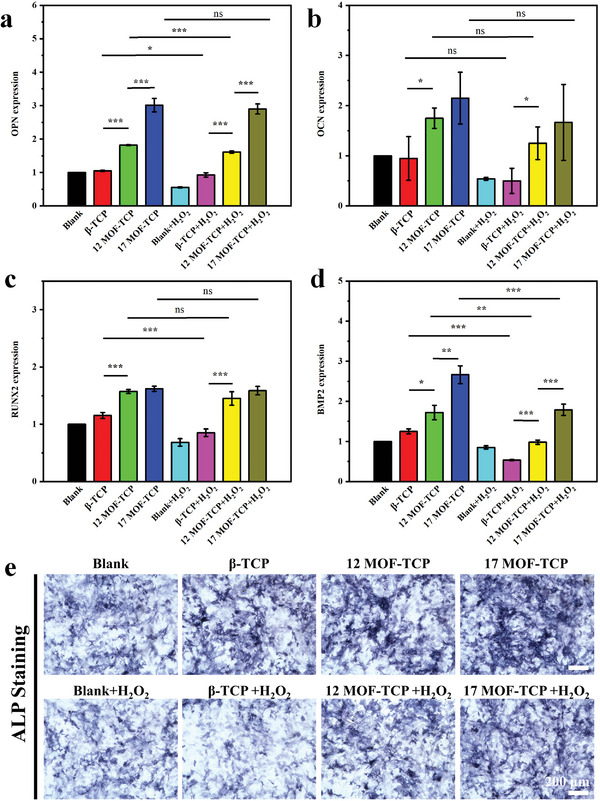
The promoting effects of MOF‐TCP scaffolds on osteogenic differentiation of rBMSCs under both normal condition and oxidative condition. The expression of a) OPN gene, b) OCN gene, c) RUNX2 gene, d) BMP2 gene of rBMSCs (*n* = 3). e) The alkaline phosphatase (ALP) staining of rBMSCs with different treatment. *p < 0.05, **p < 0.01, ***p < 0.001.

The ability of simultaneously stimulating osteogenesis and mature of cartilage is essential for the osteochondral scaffolds. To explore the dual‐bioactivity of MOF‐TCP scaffolds, their effects on the osteogenic differentiation of rBMSCs and chondrogenic differentiation of chondrocytes were investigated under both normal condition and oxidative condition. As shown in **Figure** **3**a–d, the expression of osteogenesis related genes, osteopontin (OPN), osteocalcin (OCN), runt‐related transcription factor 2 (RUNX2), and bone morphogenetic protein 2 (BMP2) of rBMSCs was evaluated by real‐time quantitative polymerase chain reaction (RT‐qPCR). In the normal culture condition, osteogenic genes expression of the rBMSCs cultured with 12MOF‐TCP and 17MOF‐TCP scaffolds was enhanced in comparison to that of the *β*‐TCP group and untreated cells (Blank). Further, osteogenic genes expression of rBMSCs was evaluated under oxidative condition which induced by H_2_O_2_ stimulation. It is obvious that 12MOF‐TCP and 17MOF‐TCP scaffolds could still effectively enhance the osteogenic differentiation of rBMSCs compared with *β*‐TCP scaffolds after H_2_O_2_ treatment. Notably, the osteogenic genes expression in Blank group and *β*‐TCP group was obviously inactivated after H_2_O_2_ treatment. In contrast, the expression of OCN and RUNX2 in 12MOF‐TCP groups showed no much difference before and after H_2_O_2_ stimulation. Furthermore, for 17MOF‐TCP group, the expression of OPN, OCN, and RUNX2 showed no significant difference under the two conditions. These results indicated that rBMSCs cultured with 12MOF‐TCP and 17MOF‐TCP were hardly hurted by H_2_O_2_. Therefore, by comparing the results under two conditions, it could be inferred that the 12MOF‐TCP and 17MOF‐TCP scaffolds protected rBMSCs from oxidative stress by scavenging H_2_O_2_. Moreover, the alkaline phosphatase (ALP) staining was conducted to visually observe the osteogenic levels of rBMSCs. As shown in Figure 3e, the color of ALP staining images was obviously darker in MOF‐TCP groups compared with that in Blank and *β*‐TCP groups. Meanwhile, although the cells under oxidative conditions produced less ALP than normal conditions, 17MOF‐TCP group was less affected by the oxidative stress and exhibited more ALP staining area.

**Figure 4:**
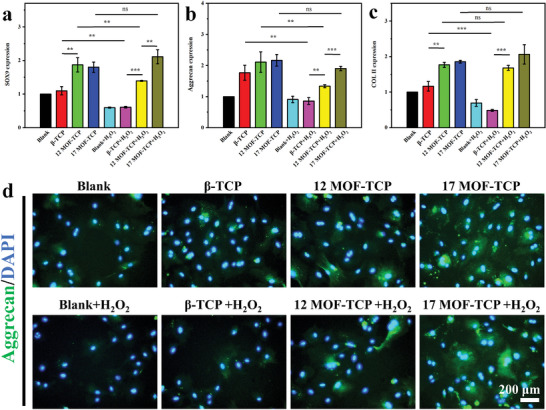
The promoting effects of MOF‐TCP scaffolds on mature of chondrocytes under both normal condition and oxidative condition. The expression of a) SOX9 gene, b) Aggrecan gene, c) COL II gene in chondrocytes (*n* = 3). d) The immunofluorescent staining of Aggrecan protein in chondrocytes with different treatment (green: Aggrecan protein, blue: cell nucleus). *p < 0.05, **p < 0.01, ***p < 0.001.

On the other hand, the expression of chondrogenesis related genes, six determing region of Y chromosome‐box transcription factor 9 (SOX9), aggrecan, and collagen II (COL II), was also evaluated. As shown in **Figure** **4**, the Aggrecan expression in *β*‐TCP, 12MOF‐TCP, and 17MOF‐TCP groups was promoted compared to Blank group. What's more, SOX9 and COL II expression of the chondrocytes cultured with 12MOF‐TCP and 17MOF‐TCP scaffolds was promoted compared with Blank and *β*‐TCP groups. After that, the genes expression of chondrocytes was further investigated under H_2_O_2_ stimulation. The gene expression of MOF‐TCP groups was still significantly higher than that of the Blank and *β*‐TCP group. Importantly, the expression of SOX9, Aggrecan, and COL II in *β*‐TCP group significantly decreased after H_2_O_2_ treatment. In contrast, the expression of these genes in 17MOF‐TCP group was comparable before and after H_2_O_2_ treatment. Besides, the expression of aggrecan protein in chondrocytes was investigated by immunofluorescent staining to evaluate the maturation levels of chondrocytes (Figure 4d and Figure S10, Supporting Information). Similar to the RT‐qPCR results, more aggrecan was produced in MOF‐TCP groups. Notably, the expression level of aggrecan protein in 17MOF‐TCP group remained almost unchanged with or without H_2_O_2_ treatment, and was significantly higher than that in the Blank and *β*‐TCP groups. By comparing the results under two conditions, we found that chondrocytes cultured with 17MOF‐TCP scaffolds were hardly affected by H_2_O_2_ stimulation. These results indicated that 17MOF‐TCP scaffolds could well protect chondrocytes from oxidative injuries by scavenging H_2_O_2_.

Overall, MOF‐TCP scaffolds, especially 17MOF‐TCP scaffolds, could significantly promote the bidirectional differentiation of cells and protect them from oxidative stress. On the one hand, MOF‐TCP scaffolds promoted the osteogenic differentiation of mesenchymal stem cells and the maturation of chondrocytes under both normal condition and oxidative condition. These promoting effects might be contributed to the bioactive ions, including Ca^2+^, (PO_4_)^3−^, Zn^2^+, and Co^2+^, released from the scaffolds. Nevertheless, *β*‐TCP scaffolds which could release Ca^2+^ and (PO_4_)^3−^ didn't show obvious promoting effects. Therefore, it could be inferred that the promotion was mainly attributed to Zn^2+^ and Co^2+^. It was reported that Zn^2+^ could effectively improve the osteogenic gene expression, ECM production, and mineral deposition by triggering the cAMP‐PKA pathway and Gaq‐PLC‐IP3 pathway.^[^ ^19^ ^]^ In addition, Co^2+^ could simultaneously promote angiogenesis and osteogenesis.^[^ ^20^ ^]^ What's more, moderate concentration of Co^2+^ was found to be anti‐inflammatory and could protect cartilage from the catabolic injury induced by IL‐1*β*.^[^ ^21^ ^]^ Accordingly, the promoting effects of MOF‐TCP scaffolds on bidirectional differentiation of rBMSCs and chondrocytes may mainly be attributed to the combination of released Zn^2+^ and Co^2+^.

Moreover, by comparing the results between normal condition and oxidative condition, we found that cell differentiation in the 12MOF‐TCP and 17MOF‐TCP groups was hardly impacted by oxidative stress, whereas that in the *β*‐TCP and Blank groups was severely inhibited by H_2_O_2_ stimulation. These results indicated that MOF‐TCP scaffolds were able to scavenge H_2_O_2_ in the culture medium to protect cells from oxidative damage while releasing ions to promote their differentiation. Taken together, the ROS‐scavenging capacity and ion release properties of Zn/Co‐MOF synergistically promoted the dual‐bioactivities of the scaffolds. Therefore, MOF‐TCP scaffolds are expected to accelerate the integrated repair of cartilage and subchondral bone in the oxidative environment.

### 2.4 MOF‐TCP Scaffolds Protected Cells from Oxidative Stress

Subsequently, in order to figure out the mechanism by which MOF‐TCP scaffolds protected cells, we fully investigated the in vitro antioxidative activities of the scaffolds. Previous studies have demonstrated that ROS levels are closely related to the inflammatory microenvironment of OA. The elevated ROS level will activate the proinflammatory signaling pathways and destroy cartilage homeostasis.^[^ ^22^ ^]^ The inflammatory cytokines, in turn, will further stimulate the generation of multiple ROS.^[^ ^23^ ^]^ Besides, the intra‐articular infiltration of inflammatory cells, especially macrophages, was formed in response to excessive ROS and closely associated with the pathogenesis of OA.^[^ ^24^ ^]^ Therefore, we comprehensively analyzed the regulatory effects of MOF‐TCP scaffolds on ROS levels and proinflammatory cytokines of multiple cells, including mesenchymal stem cells, chondrocytes, and macrophages.

**Figure 5:**
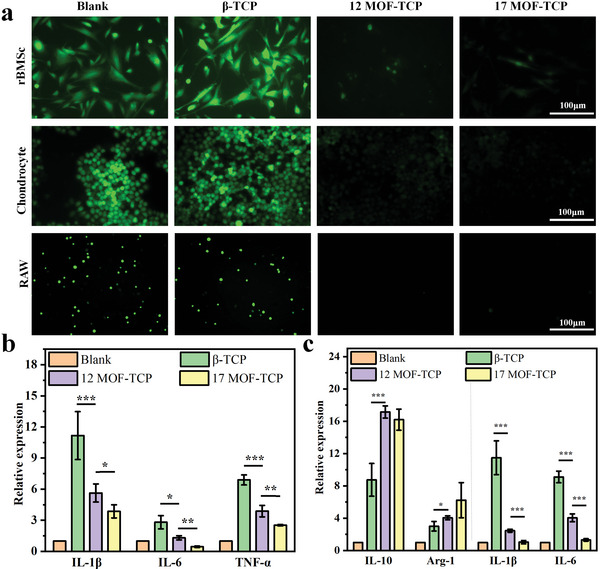
In vitro antioxidative and anti‐inflammatory activities of MOF‐TCP scaffolds. a) ROS fluorescence staining images in rBMSCs, chondrocytes, and RAW 264.7 cells cultured with scaffolds under H_2_O_2_ stimulation. b) The expression of proinflammatory genes in chondrocytes (*n* = 3). c) The expression of anti‐inflammatory genes (IL‐10 and Arg‐1) and proinflammatory genes (IL‐1*β* and IL‐6) in RAW 264.7 cells (*n* = 3). *p < 0.05, **p < 0.01, ***p < 0.001.

To investigate the inhibited effects of MOF‐TCP scaffolds on intracellular ROS level, the cells were incubated with multiple ROS, including H_2_O_2_, Rosup, O_2_^•−^, ^•^OH, and ONOO^−^, to build different oxidative microenvironment. Then the cellular ROS levels of rBMSCs, chondrocytes, and macrophages (RAW246.7 cells) were monitored by a ROS indicator 2′,7′‐dichloro‐fluorescein diacetate (DCFH‐DA). As shown in **Figure** **5**a, the green fluorescence intensity represented the cellular ROS level. High fluorescence signals were monitored upon H_2_O_2_ stimulation in the Blank group, indicating that high level of external ROS would intrude into the cells and make intracellular ROS increasing. In addition, the treatment of *β*‐TCP scaffolds could not prevent the external ROS from invading the cells. However, the fluorescence intensity dramatically decreased in the MOF‐TCP groups, demonstrating that the MOF‐TCP scaffolds presented excellent antioxidative activity of scavenging the external H_2_O_2_. Furthermore, the antioxidative effects of MOF‐TCP scaffolds on scavenging other types of ROS were investigated. Figures S7–S9 (Supporting Information) exhibit the ROS levels in rBMSCs, chondrocytes, and macrophages, respectively, under Rosup, O_2_^•−^, ^•^OH, and ONOO^−^ stimulation. Similarly, MOF‐TCP scaffolds hindered these ROS intruding into the cells and prevented the cells from oxidative stress.

To investigate the inflammatory regulation effects of MOF‐TCP scaffolds, the inflammatory gene expression of chondrocytes and RAW 246.7 cells was analyzed. Compared with untreated cells (Blank), chondrocytes treated with H_2_O_2_ and scaffolds showed upregulated expression of inflammatory genes (Figure 5b), verifying that the oxidative environment was awfully proinflammatory. However, the MOF‐TCP scaffolds, especially 17MOF‐TCP scaffolds, significantly inhibited the expression of inflammatory factors in comparison with *β*‐TCP scaffolds. This result indicated that MOF‐TCP scaffolds could protect chondrocytes from inflammatory damages caused by ROS‐excessive environment. Moreover, the expression of anti‐inflammatory genes (IL‐10, Arg‐1) and proinflammatory genes (IL‐1*β*, IL‐6) in RAW264.7 cells was further investigated (Figure 5c). After treatment of MOF‐TCP scaffolds, the proinflammatory genes expression in RAW cells was downregulated and anti‐inflammatory genes expression was upregulated. It could be inferred that MOF‐TCP scaffolds possessed the inflammatory regulation ability and could mediate the immune environment under oxidative conditions.

In a word, these results demonstrated that MOF‐TCP scaffolds were able to protect cells from excessive ROS in the microenvironment and mediate an anti‐inflammatory environment, both of which are critical in treating OA. It is reported that the overproduced ROS, such as O_2_^•−^, H_2_O_2_, and ONOO^−^, in the joint cavity would hurt the chondrocytes by causing hyperperoxidation, protein carbonylation, and DNA damage.^[^ ^25^ ^]^ Meanwhile, the inflammatory environment closely related with excessive ROS would accelerate the pathological process of OA and hinder the formation of new tissues.^[^ ^2^ ^]^ We may conclude that MOF‐TCP scaffolds scavenged ROS by the functionalized Zn/Co‐MOF, thus protecting cells from oxidative stress and inflammation. Interestingly, the fluorescence images of intracellular ROS analysis showed that 12MOF‐TCP and 17MOF‐TCP scaffolds exhibited similar degree of in vitro ROS‐scavenging capacity. However, 17MOF‐TCP scaffolds showed better inflammatory regulation effects than 12MOF‐TCP scaffolds. This might be due to the higher concentration of Zn^2+^ released by 17MOF‐TCP. As shown in Figure S3 (Supporting Information), few Zn^2+^ were released from the 12MOF‐TCP scaffolds, while the 17MOF‐TCP scaffolds released significantly more Zn^2+^. Studies have demonstrated that appropriate dose of Zn^2+^ could induce and maintain an anti‐inflammatory microenvironment by activating mTOR signaling pathways.^[^ ^26^ ^]^ Therefore, taking advantages of the antioxidative activities and the released bioactive ions, 17MOF‐TCP scaffolds are promising to promote the osteochondral regeneration under OA conditions by scavenging excessive ROS and mediating anti‐inflammatory microenvironment. With the best dual‐bioactivities as well as antioxidative capacity, 17MOF‐TCP scaffolds were optimized for the further in vivo study.

### 2.5 MOF‐TCP Scaffolds Promoted In Vivo Osteochondral Regeneration

**Figure 6:**
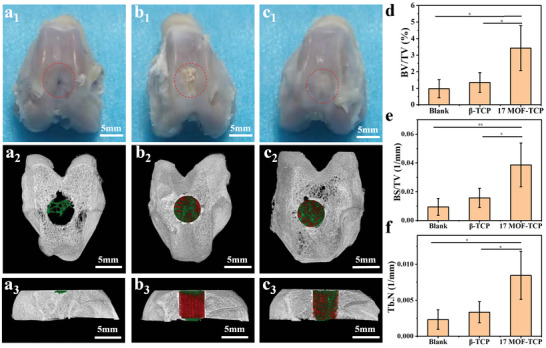
The in vivo regeneration effects of the osteochondral defects treated with different scaffolds. Digital photographs (a1–c1), the transverse view images (a2–c2), and sagittal view images (a3–c3) of the osteochondral defects reconstructed by microcomputed tomography (Micro‐CT). The green, red, and gray‐white color in Micro‐CT images represent new bone, scaffold, and native bone, respectively. The newly formed bone assessment including d) the ratio of new bone volume to defect volume (BV/TV), e) bone surface mineral density (BS/TV), f) the trabecular number of the new bone (Tb. N) after 12 weeks of implantation (*n* = 5). *p < 0.05, **p < 0.01, ***p < 0.001.

In order to fully evaluate the in vivo regenerative efficacy of MOF‐TCP scaffolds, the scaffolds were implanted into the rabbit osteochondral defects. Three groups were investigated, the 17MOF‐TCP group, the *β*‐TCP group, and the Blank group without implantation. After 12 weeks of implantation, all rabbit joint samples were harvested. Firstly, Micro‐CT reconstruction was conducted to analyze the regenerative efficiency of subchondral bone. As shown in **Figure** **6**, few new tissues were formed in the Blank groups, indicating the poor self‐healing ability of osteochondral tissues. According to the transverse view of Micro‐CT images, much more calcified tissue could be observed in the *β*‐TCP and 17MOF‐TCP groups, while a large vacancy remained in the Blank group. Besides, sagittal views indicated that new tissues were only generated in surface area of the defects in Blank group. On the contrary, many new bone tissues were generated in the margin of implanted scaffolds. Furthermore, much more new bone tissues were abundant and evenly generated in the interspace of 17MOF‐TCP groups (Figure 6a–c). Notably, the matrix of the scaffolds was partly degraded and replaced by the newly formed bone, indicating that the scaffolds could gradually degrade and support the growth of new bone.

Furthermore, the relative bone volume fraction (BV/TV), the bone surface mineral density (BS/TV), and the trabecula number (Tb. N) of the defects were calculated to analyze the new bone tissues. According to the statistical results, 17MOF‐TCP group significantly regenerated more new bone tissues (Figure 6d–f). To further investigate the bone regenerative efficacy, all samples were sectioned and stained by safranine O/Fast green and Van Gieson's picrofuchsin (VG). As shown in Figure S10 (Supporting Information), there were still apparent cavities in the defects of Blank group. In the *β*‐TCP group, more subchondral bone tissues were formed compared to the Blank group, but the new tissues could not fulfill the defects. In the 17MOF‐TCP group, large amount of subchondral bone was formed and tightly integrated with the 17MOF‐TCP scaffolds, and the calcified cartilage layer could be obviously observed. Taken together, these results indicated that the MOF‐TCP scaffolds could significantly promote the regeneration of subchondral bone.

**Figure 7:**
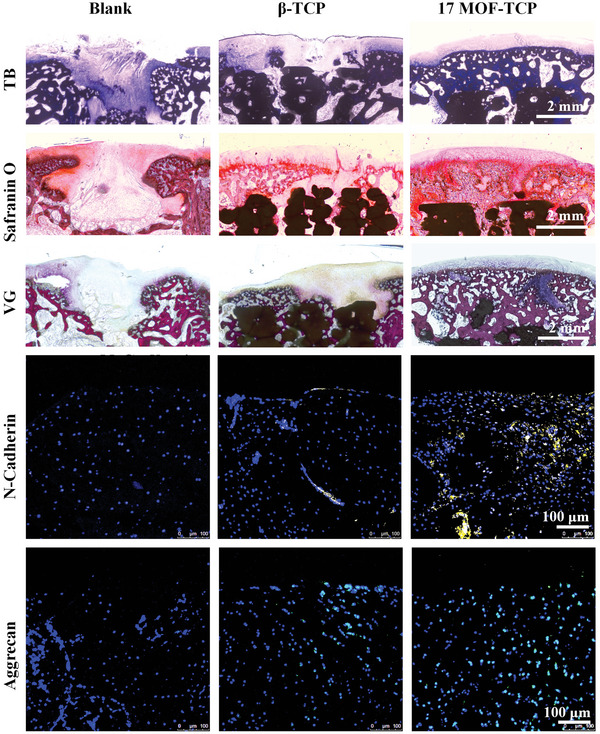
Histological analysis of the in vivo regeneration of the osteochondral defects. Toluidine blue (TB) staining images, Safranin O staining images, Van Gieson staining images, N‐Cadherin fluorescence staining images, and Aggrecan fluorescence staining images of the defects after 12 weeks of implantation.

To evaluate the efficacy of MOF‐TCP scaffolds for hyaline cartilage regeneration, further histological analysis was performed using toluidine blue (TB) staining, safranine O staining, and VG staining. Shown in **Figure** **7**, the defects of Blank group were covered with fibrous tissue. Compared to Blank group, there were more cartilage tissues formed in the *β*‐TCP group. However, the new cartilage tissues did not fulfill the defects and did not show the similar characteristics to natural cartilage. Encouragingly, new hyaline cartilage which possessed abundant ECM and lots of mature chondrocytes was fully formed in the 17MOF‐TCP group. Furthermore, the orderly continuous osteochondral interface was formed in the 17MOF‐TCP group. Then, the cartilage regeneration levels were quantitatively evaluated by the O'Driscoll grading system. As shown in Figure S15 (Supporting Information), the average score 8.3 ± 2.11 in Blank group, 12.3 ± 2.58 in *β*‐TCP group and 19.7 ± 2.00 in 17MOF‐TCP group, respectively. In addition, immunofluorescence staining assays of N‐Cadherin and Aggrecan were conducted to investigate the quality of newly regenerated cartilage. As shown in Figure 7, it is obvious that more N‐Cadherin and Aggrecan were expressed in the 17MOF‐TCP group. These analysis results further demonstrated that 17MOF‐TCP scaffolds distinctly facilitated hyaline cartilage regeneration.

Therefore, these results showed that 17MOF‐TCP scaffolds significantly accelerated the regeneration of cartilage and subchondral bone. On the one hand, as mentioned above, Zn^2+^ and Co^2+^ released from 17MOF‐TCP scaffolds could promote the regeneration of cartilage and subchondral bone as well as their interface. On the other hand, the excellent antioxidative activity of 17MOF‐TCP scaffolds enables them to regulate inflammatory environment and inhibit ROS overproduction in the acute inflammatory condition caused by drilling the defects. Thus, 17MOF‐TCP scaffolds created a suitable microenvironment for regeneration of new tissues. For these reasons, 17MOF‐TCP scaffolds possessed the ideal function of promoting the osteochondral regeneration in vivo.

## 3 Conclusion

In summary, by moderately functionalizing Zn/Co‐MOF on *β*‐TCP scaffolds, we successfully developed a multifunctional scaffold (MOF‐TCP) with ROS‐scavenging, inflammatory regulation, and osteochondral regeneration capabilities to repair osteochondral defects. By modulating concentration of Zn/Co‐MOF reaction solution, the MOF‐TCP scaffolds exhibited broad‐spectrum ROS‐scavenging activities and satisfactory biocompatibility. Interestingly, MOF‐TCP scaffolds could simultaneously enhance osteogenic differentiation of rBMSCs and mature of chondrocytes, as well as protect them from oxidative stress by eliminating external ROS and mediating anti‐inflammatory microenvironment. As a result, MOF‐TCP scaffolds showed remarkable promoting effects on integrated regeneration of osteochondral tissues in vivo. Hence, such novel osteochondral scaffolds with wonderful antioxidative capability show great potential for the treatment of OA, and the strategy proposed by this study may provide a new solution to repair tissue defects caused by inflammatory disease.

## 4 Experimental Section

### Materials

The *β*‐tricalcium phosphate (*β*‐TCP) was purchased from Kunshan Overseas Chinese Technology New Material Co., Ltd (Kunshan, China). The pluronic F‐127 was purchased from Sigma–Aldrich (USA). The Zn(NO_3_)_2_·6H_2_O, Co(NO_3_)_2_·6H_2_O, and 2‐methylimidazole (2‐MIM) were purchased from Shanghai Aladdin Biochemical Technology Co., Ltd (Shanghai, China).

### Preparation and Characterization of MOF‐TCP Scaffolds

Firstly, pure *β*‐TCP scaffolds were fabricated by 3D printing. Typically, 10 g of *β*‐TCP powders and 0.06 g of sodium alginate were added into the F‐127 solution with a concentration of 20 wt%. Then the obtained mixture was stirred and transferred into cartridge for next printing process. After that, cylindrical scaffolds were 3D printed with the extrusion pressure in the range of 300 kPa to 600 kPa. The printed green scaffolds were then dried and sintered at 1150 °C for 3 h to obtain the ultimate *β*‐TCP scaffolds.

To deposit Zn/Co‐MOF on the scaffolds, 3D‐printed *β*‐TCP scaffolds were reacted with different concentration of Zn/Co‐MOF reaction solution. Firstly, 2.87 g of 2‐MIM was dissolved in 100 mL H_2_O under magnetic stirring to obtain A solution (0.35 mol L^−1^). Meanwhile, Zn(NO_3_)_2_·6H_2_O and Co(NO_3_)_2_·6H_2_O with a molar ratio of 1:1 was dissolved into 15 mL H_2_O to form B solution with a total concentration of Zn/Co ions at 0.5 mmol L^−1^. After that, *β*‐TCP scaffolds were immersed into the A solution with different diluted concentration. Subsequently, B solution with different diluted concentration were dropwise added into them for further reaction. The proportions of A and B solution in the Zn/Co‐MOF reaction solution for preparing different MOF‐TCP scaffolds were listed in Table S1 (Supporting Information). After reaction at room temperature for 3 h, the MOF‐TCP scaffolds with different amount of Zn/Co‐MOF were obtained.

The interior structure of the scaffolds (17MOF‐TCP scaffolds with diameter of 7.5 ± 0.5 mm and height of 10 ± 0.5 mm) was reconstructed by micro‐CT (Bruker, Germany). And, the total porosity of the 17MOF‐TCP scaffolds was calculated based on the 3D‐reconstructed structure via CTAn software (Bruker). The microscopic morphologies of the 3D‐printed scaffolds were observed by using a SEM (S‐4800, Hitachi, Japan). Meanwhile, the elemental distribution of MOF‐TCP scaffolds was detected by the energy‐dispersive spectrometer. The phase compositions of *β*‐TCP and MOF‐TCP scaffolds were determined ​by an X‐ray diffractometer (Bruker). The compressive strength of scaffolds was tested at a constant compression rate of 0.5 mm min^−1^ by a static mechanical test machine (INSTRON 5566, Germany). To investigate the ion release properties of the scaffolds, the scaffolds were immersed into the Tris‐HCl solutions for 1, 3, 5, 7, and 14 days. At each time point, the Tris–HCl solution was harvested and the Ca, P, Co, and Zn ions concentration in the solution was detected by an inductively coupled plasma atomic emission spectroscopy (ICP–AES, 715‐ES, Varian, America).

### ROS‐Scavenging Capability of MOF‐TCP Scaffolds

The antioxidative activity of MOF‐TCP scaffolds was evaluated by a multifunction microplate reader (SpectraFluor Plus, Tecan, Crailsheim, Germany). First, ABTS and DPPH free radicals were used to investigate the ROS‐scavenging capability of MOF‐TCP scaffolds. Briefly, ABTS (0.007 mol L^−1^) and potassium persulfate (0.005 mol L^−1^) were mixed with a volume ratio of 1: 1 and reacted in the dark for 24 h. Then, scaffolds were immersed into the ABTS working solution. After reacted for 3 h, the absorbance of the solution at 730 nm was recorded by a multifunction microplate reader. Besides, DPPH working solution was prepared by adding 0.002 g of DPPH into 50 mL of dehydrated alcohol. Then, the scaffolds were immersed into the DPPH working solution for 72 h and their absorbance at 520 nm was measured.

The ability of the scaffolds to scavenge ONOO^‐^ was investigated as follows. First, NaNO_2_ solution (15 mL; 50 mmol L^−1^) and H_2_O_2_ (15 mL; 25 mmol L^−1^) were added into a 50 mL beaker and stirred for 5 min. Next, HCl (7.5 mL; 1 mol L^−1^) and NaOH (7.5 mL; 1.5 mol L^−1^) were added and the mixture were stirred until its appearance changed from transparent to light yellow. In order to ensure the consistency of experiment conditions, the mixture solution, of which absorbance at 302 nm was equal to 0.9, was set as the standard working solution. Therefore, the original mixture solution was diluted two to four times to meet this requirement. Subsequently, the scaffolds were reacted with the ONOO^−^ working solution for 1 h and recorded their absorbance at 302 nm.

SOD‐like activity of MOF‐TCP scaffolds was determined by a total SOD kit with WST‐8 (S0101S, Beyotime, China). Briefly, following the manufacturer's protocol, the SOD working solution was prepared and reacted with the scaffolds for 0.5 h, and the absorbance at 460 nm of the solution was measured.

CAT‐like activity of MOF‐TCP scaffolds was evaluated via two methods. First, H_2_O_2_ content detection kit (BC3595, Solarbio, China) was used to measure CAT‐like activity. Following the manufacturer's protocol, the CAT working solution was prepared, and then reacted with the scaffolds for 6 h. After that, the scaffolds were taken out from the CAT solution. And, an equivalent chromogenic reagent was added to the solution at room temperature for 5 min. Finally, the absorbance of the final solution at 415 nm was measured. Second, a portable dissolved oxygen tester (JPB‐607A, Leici, Shanghai Yidian Scientific Instrument Co., Ltd, China) was applied to measure the production of oxygen in the H_2_O_2_ scavenging process. Briefly, a scaffold was added into H_2_O_2_ solution (100 mmol L^−1^) and the dissolved oxygen was detected by the portable dissolved oxygen tester in 10 min.

Finally, the elimination ratios of multiple ROS were calculated according to the following formula: Elimination ratio (%) = [(*A*_0 −_ *A*)/*A*_0_] ×100. *A*_0_ represented the absorbance value of the ROS working solution without scaffolds added. *A* represented the absorbance value of the ROS solution after reacted with different scaffolds.

### Cell Culture

Primary bone marrow mesenchymal stem cells (rBMSCs) and chondrocytes were isolated from 3‐week‐old New Zealand white rabbits. The murine‐derived macrophage cell line RAW 264.7 was purchased from the cell bank of the Chinese Academy of Sciences. The minimum Essential Medium‐*α* (MEM‐*α*, Gibco, USA), which was supplemented with 10% fetal bovine serum (FBS, Gibco) and 1% penicillin‐streptomycin (P/S, Gibco), was applied to culture rBMSCs. Meanwhile, the high‐glucose Dulbecco's modified Eagle medium (DMEM, Sangon, China), supplemented with 10% FBS (Gibco) and 1% penicillin‐streptomycin (P/S, Gibco), was used for the culture of chondrocytes and RAW 264.7 cells. All cells were cultured at 37 °C in a humidified incubator supplemented with 5% CO_2_.

### Cytocompatibility of the Scaffolds

The scaffolds prepared by different concentration of Zn/Co‐MOF reaction solution (6.25–50 × 10^−3^ m) were seed with rBMSCs (5 × 10^4^ cells per scaffold) in 48‐well plates and cultured for 24 h. Then, the rBMSCs with scaffolds were incubated in Cell Counting Kit‐8 (CCK‐8, Dojindo, Japan) working solution for 1 h. The absorbance at 450 nm of the incubation solution was evaluated to calculate the cell viability. Notably, viability of the rBMSCs cultured on the *β*‐TCP scaffolds was considered as 100%. The cell viability was calculated based on the following formula: Cell viability (%) = *C*/*C*_0_ × 100. *C*_0_ represented the absorbance value of the cells cultured with pure *β*‐TCP scaffolds. *C* represented the absorbance value of the rBMSCs cultured with different scaffolds.

### Proliferation and Adhesion of rBMSCs and Chondrocytes on the Scaffolds

rBMSCs (5 × 10^4^ cells per scaffold) and chondrocytes (2.5 × 10^5^ cells per scaffold) were seeded on the scaffolds in 48‐well plates and cultured for 1, 3, 5, and 7 days to evaluate their proliferation activities. At each time point, the scaffolds with cells were incubated in 10% CCK‐8 solution for 1 h. Subsequently, the absorbance at 450 nm of the incubation medium was measured. The absorbance value was regarded as the quantity of cells. In addition, to evaluate the cell adhesion, rBMSCs and chondrocytes were cultured on the scaffolds for 24 h. Then, all samples were fixed with 4% paraformaldehyde and the cell morphologies were captured by confocal laser scanning microscopy (CLSM, TCS SP8, Leica, Germany). Diamidinophenylindole (DAPI, Sigma–Aldrich) and rhodamine‐phalloidin (Amyjet Scientific, Wuhan, China) marked the nuclei and cytoskeletons of the cells, respectively. The cell adhesion numbers were analyzed by calculating cell numbers in three randomly selected magnified fields via Image J software.

### Migration Activities of rBMSCs and Chondrocytes under Treatment of the Scaffolds

First, rBMSCs and chondrocytes were seeded in the 6‐well plates with a density of 2 × 10^5^ cells per well. When the cells proliferated to cover the well, a scratch was made in the middle of each well by using the 1 mL pipette tip. The optical images of the cell samples were captured as soon as the scratches were created. After that, the scaffolds were put into the Transwell and cocultured with the cells. Meanwhile, the medium was changed by the H_2_O_2_‐containing medium (200 µmol L^−1^). In addition, the medium only contained 1% serum to avoid the proliferation of cells. After incubation for 24 h, the cell samples were fixed with the 4% paraformaldehyde and stained by the 0.1% crystal violet solution. Then, their images were captured and analyzed by Image J software. By measuring the area of scratches, the migration ratio could be calculated.

### Specific Differentiation of rBMSCs and Chondrocytes on the Scaffolds

Osteogenic genes of rBMSCs and chondrogenic genes of chondrocytes were investigated by RT‐qPCR. Firstly, the cells were seeded on the scaffolds with the density of 5 × 10^4^ rBMSCs per scaffold and 2.5 × 10^5^ chondrocytes per scaffold. After 7 days of culture, cells on the scaffolds were dissociated by TRIZOL reagent. Then, their total RNA was extracted and reverse transcribed to cDNA by using a ToYoBo RNAprep Micro Kit (FSK 201, ToYoBo, Japan). After that, RT‐qPCR processes were conducted by using an SYBR Green qPCR Master Mix (QPK‐201, ToYoBo) via a Step One Plus Real‐Time PCR system (Thermofisher, USA). After the RT‐qPCR cycle operation was done, 2^−ΔΔCt^ method was used to analyze the relative gene expression. GAPDH was employed as a reference gene and the primer sequences of all genes were shown in Tables S2 and S3 (Supporting Information).

For the ALP staining of rBMSCs, the rBMSCs were seeded on the 12‐well plates with the density of 2 × 10^4^ rBMSCs per well. Then, the scaffolds were put into the Transwell and cocultured with the cells for 7 days under H_2_O_2_ stimulation (200 µmol L^−1^). After that, the cells were anchored by 4% paraformaldehyde and then stained by a BCIP/NBT Alkaline Phosphatase Color Development Kit (C3206, Beyotime). The staining images were captured by the microscope (DMI8, Leica).

For the immunofluorescent staining of aggrecan in chondrocytes, the chondrocytes were seeded on the 12‐well plates with the density of 2 × 10^4^ chondrocytes per well. Then, the scaffolds were put into the Transwell and cocultured with the cells for 7 days under H_2_O_2_ stimulation (200 µmol L^−1^). After that, the cells were anchored by 4% paraformaldehyde for 24 h and washed by PBS. Then, the cells were incubated with the block solution (P0260, Beyotime) for 30 min and incubated with the primary antibody working solution (sc25674, Santa Cruz, USA) at 4 °C overnight. On the second day, the cells were washed by PBS for three times and incubated with the second antibody working solution (ab150077, Abcam, USA) at 20 °C for 1 h. DAPI (Sigma–Aldrich) was used to stain the nucleus. After that, the cells were washed by PBS for three times and observed by fluorescence microscope (DMI8, Leica).

### In Vitro Antioxidative Activities of the Scaffolds

To investigate the in vitro antioxidative activities of MOF‐TCP scaffolds, intercellular ROS were detected using DCFH‐DA (S0033, Beyotime). DCFH‐DA is a nonfluorescent compound which can react with intracellular ROS and generate the fluorescent product of dichloro‐fluorescein (DCF). As a result, the intensity of the fluorescence produced by DCF is positively correlated with the amount of intracellular ROS. First, cells were seeded on the culture plate and cultured for 24 h. Then, oxidative stress was induced by exposing the cells to the culture medium with ROSup (50 ug mL^−1^, 12 h), H_2_O_2_ (500 µmol L^−1^, 2 h), FeSO_4_ and H_2_O_2_ (500 µmol L^−1^, 2 h), O_2_^•−^ (1 h), and ONOO^−^ (0.5 h), respectively. The O_2_^•−^ working solution was prepared following the manufacturer's protocol (S0101S, Beyotime). The ONOO^−^ working solution was prepared by diluting ONOO^−^ stock solution for 25 times by culture medium. Simultaneously, the cells were treated by the scaffolds by putting the Transwell chamber containing scaffolds onto the cells. After the incubation, the culture medium and scaffolds were removed, and then the cells were incubated with a DCFH‐DA probe. Finally, fluorescent images were captured using an inverted fluorescence microscope (DMI8, Leica).

To investigate the anti‐inflammatory properties of MOF‐TCP scaffolds, the inflammation‐related genes were evaluated. The untreated cells (chondrocytes and RAW 264.7 cells) were set as the Blank control group. Meanwhile, the *β*‐TCP, 12 MOF‐TCP, and 17 MOF‐TCP groups represented the cells that treated by H_2_O_2_ (200 µmol L^−1^) and incubated with *β*‐TCP, 12 MOF‐TCP, and 17 MOF‐TCP scaffolds, respectively. After chondrocytes were incubated with scaffolds for 4 days, anti‐inflammatory genes (IL‐1 *β*, IL‐6, and TNF‐ *α*) were analyzed by RT‐qPCR according to the methods mentioned above. Furthermore, the related genes in RAW 264.7 (IL‐10, Arg‐1, IL‐1*β*, and IL‐6) were measured after RAW 264.7 cells were incubated with scaffolds for 2 days. The primer sequences of these genes were shown in Tables S3 and S4 (Supporting Information).

### In Vivo Osteochondral Regeneration

An osteochondral defect model was built to evaluate the osteochondral regeneration ability of MOF‐TCP scaffolds in vivo. All rabbits were treated according to the guidelines of Institutional Animal Care and Utilization Committee of Nanjing First Hospital, Nanjing Medical University (DWSY‐2102466). New Zealand white rabbits (≈2.5 kg) were used to fabricate osteochondral defects (diameter: 5 mm, height: 5 mm) and implanted with the *β*‐TCP or 17MOF‐TCP scaffolds. The defects without any implantation were regarded as the blank group (*n* = 6). After implantation for 12 weeks, knee samples were harvested and then fixed in 4% paraformaldehyde solution for further evaluation.

All the samples were scanned using Micro‐CT (Skyscan1172, Bruker). The transverse view and sagittal view of the defects were 3D reconstructed by the CT vox short cut software. The bone volume/total volume (BV/TV) value, the bone surface mineral density (BS/TV), and the trabecula number (Tb. N) were analyzed by CT‐Analyzer program. In addition, the samples were embedded in polymethylmethacrylate and cut into sections for histological analysis. The polished tissue sections were stained by safranin‐O/fast green, improved VG, safranin‐O, and TB to further evaluate the osteochondral regeneration efficiency. For quantitatively analysis, all sections were blindly scored by five persons based on the O'Driscoll grading system.^[^ ^27^ ^]^ Furthermore, the samples were decalcified with 10% EDTA solution for 1 month, and then were embedded in paraffin wax and sectioned. After that, immunofluorescence staining of N‐Cadherin and Aggrecan was performed to evaluate the quality of regenerated cartilage. The slices were stained with primary antibodies (N‐Cadherin and Aggrecan) overnight at 4 °C. Subsequently, they were further stained with second antibodies conjugated with fluorescent dyes and DAPI (1:1000). Finally, the immunofluorescence images were recorded by CLSM.

### Statistical Analysis

All results were expressed as means ± standard deviation (SD). All data were analyzed by the Origin 2017 software (OriginLab, USA). The sample size (*n*) for each statistical analysis was indicated in corresponding figure legends. The significant differences between groups were assessed using one‐way ANOVA with a post hoc test. A *p*‐value < 0.05 was considered statistically significant and the data were indicated with (*) for probability of less than 0.05 (*p* < 0.05), **p < 0.01, and ***p < 0.001.

## Conflict of Interest

The authors declare no conflict of interest.

## Supporting information

Supporting Information

## Data Availability

The data that support the findings of this study are available from the corresponding author upon reasonable request.
