# Supplementary material for: Metal‐Organic Framework Functionalized Bioceramic Scaffolds with Antioxidative Activity for Enhanced Osteochondral Regeneration
Source: Adv Sci (Weinh). 2023 Feb 24;10(13):2206875. doi: 10.1002/advs.202206875 (PMC10161093; doi:10.1002/advs.202206875)
Supplement: Supplementary file 1 — Supporting Information [file ADVS-10-2206875-s001.pdf]

ADVANCED SCIENCE
Open Access

Supporting Information

for *Adv. Sci.*, DOI 10.1002/advs.202206875

Metal-Organic Framework Functionalized Bioceramic Scaffolds with Antioxidative Activity for Enhanced Osteochondral Regeneration

Chaoqin Shu, Chen Qin, Lei Chen, Yufeng Wang, Zhe Shi, Jiangming Yu, Jimin Huang, Chaoqian Zhao, Zhiguang Huan, Chengtie Wu, Min Zhu* and Yufang Zhu**

Supporting Information

Metal-organic Framework (MOF) Functionalized Bioceramic Scaffolds with Antioxidative Activity for Enhanced Osteochondral Regeneration

Chaoqin Shu[1 #], Chen Qin[1 #], Lei Chen[1], Yufeng Wang[1], Zhe Shi[1], Jiangming Yu[2], Jimin Huang[1,4], Chaoqian Zhao[1], Zhiguang Huan[1,4], Chengtie Wu[1,4], Min Zhu[3]*, Yufang Zhu[1,4]**

[1]*State Key Laboratory of High Performance Ceramics and Superfine Microstructure, Shanghai Institute of Ceramics, Chinese Academy of Sciences, Shanghai 200050, P. R. China.*

[2]*Department of Orthopaedics, Tongren Hospital, Shanghai Jiaotong University, Shanghai, 200336, P. R. China*

[3]*School of Materials and Chemistry, University of Shanghai for Science & Technology, Shanghai 200093, P. R. China.*

[4]*Center of Materials Science and Optoelectronics Engineering, University of Chinese Academy of Sciences, Beijing 100049, P. R. China.*

[#] These authors contributed equally to this work.

[*]Corresponding author.

Prof. Jiangming Yu, E-mail: yjm_st@163.com

Prof. Min Zhu, Email: mzhu@usst.edu.cn

Prof. Yufang Zhu, Email: zjf2412@163.com

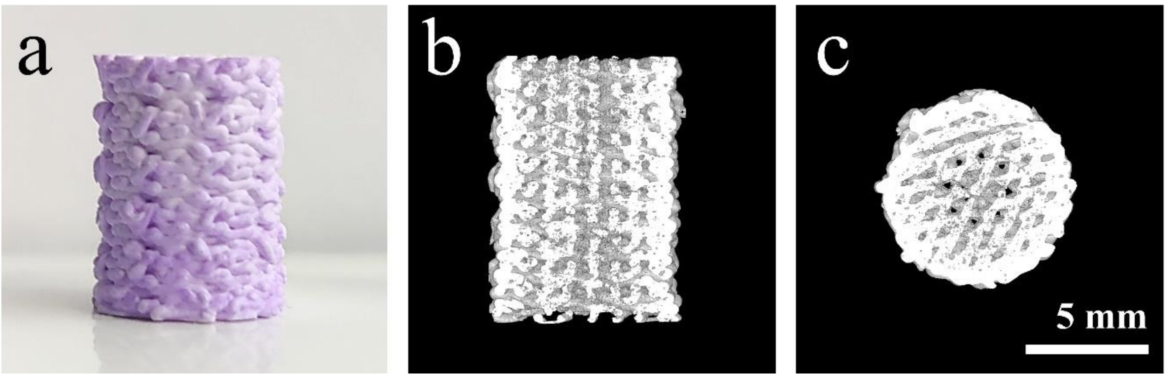

Figure S1. (a) Digital photo of the larger-sized 17MOF-TCP scaffold. Vertical-section view (b) and cross-section view (c) of the 3D reconstructed scaffold.

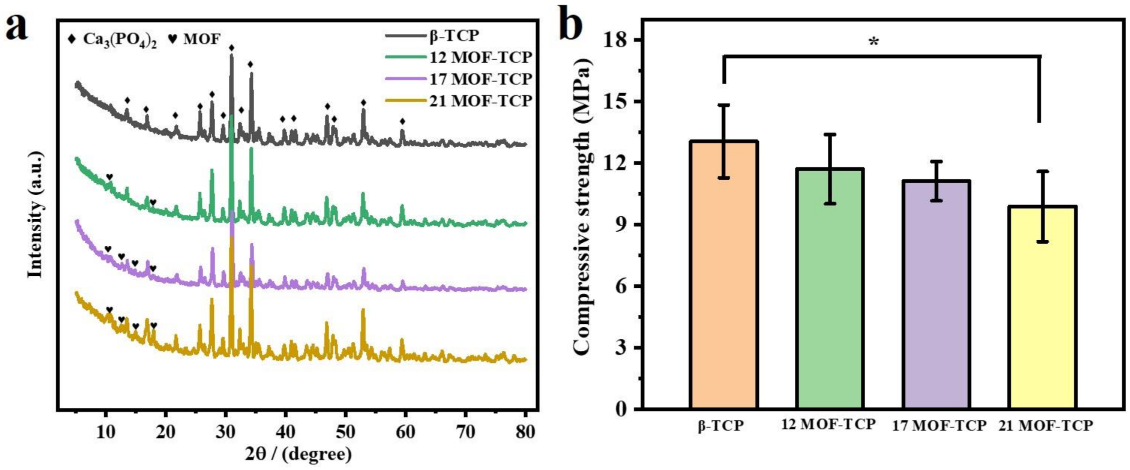

Figure S2. (a) The XRD patterns and (b) the compressive strength of the scaffolds functionalized with different concentrations of Zn/Co-MOF reaction solution (n=8). *$p < 0.05$

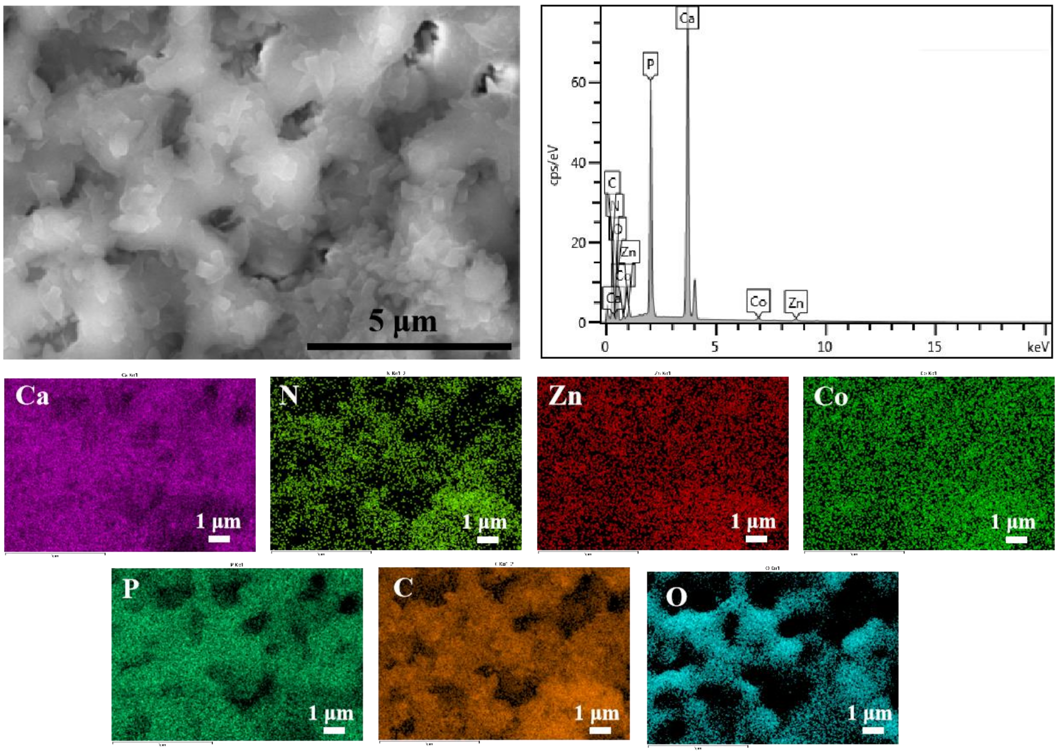

Figure S3. Element distribution on the surface of 17MOF-TCP scaffolds.

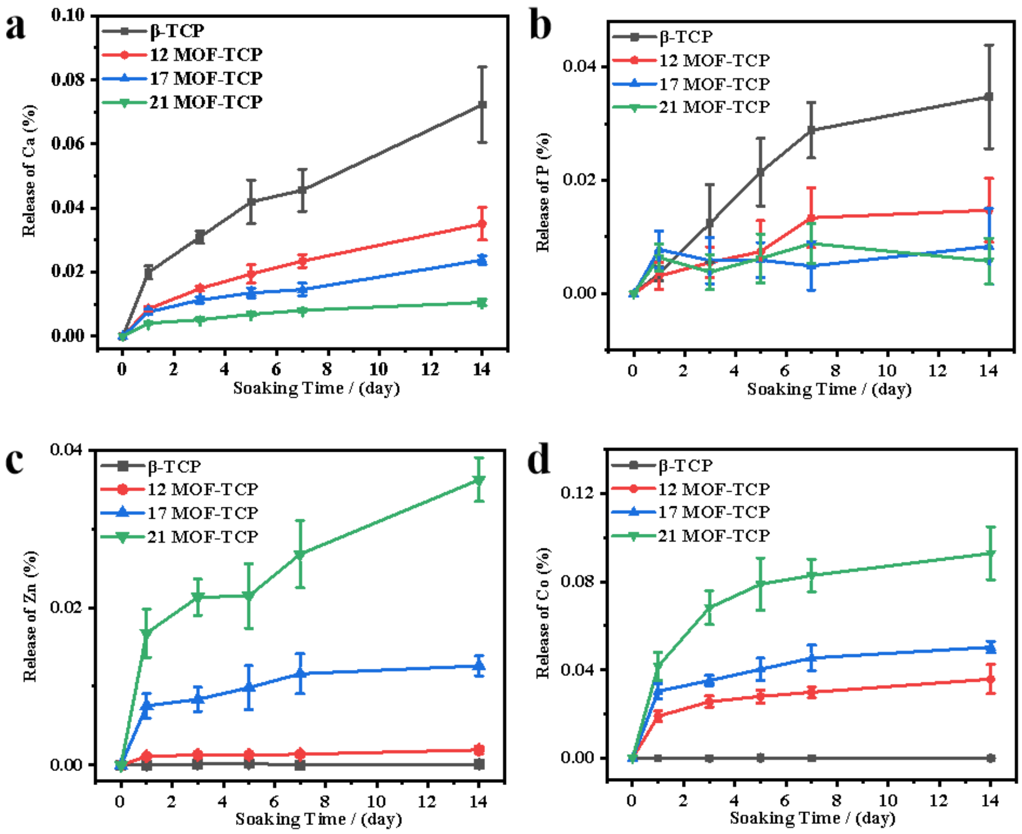

Figure S4. The release profiles of (a) Ca ions, (b) P ions, (c) Zn ions, and (d) Co ions from MOF-TCP scaffolds in Tris–HCl solution (n=5).

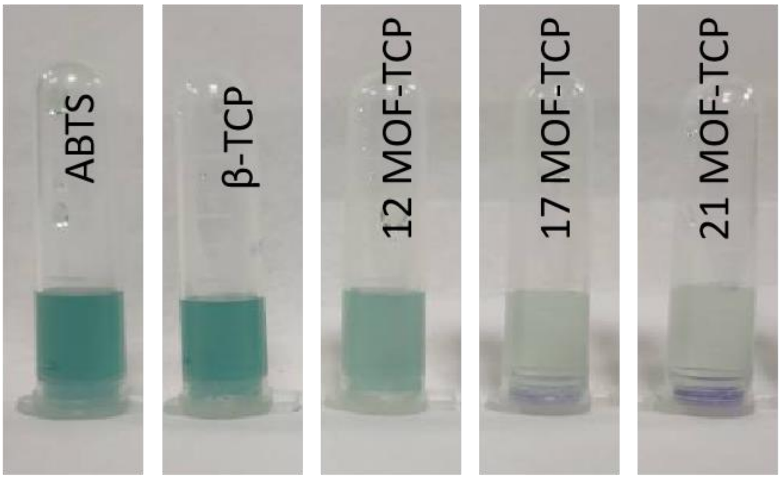

Figure S5. The color changes of ABTS free radicals after reacted with the β-TCP and MOF-TCP scaffolds.

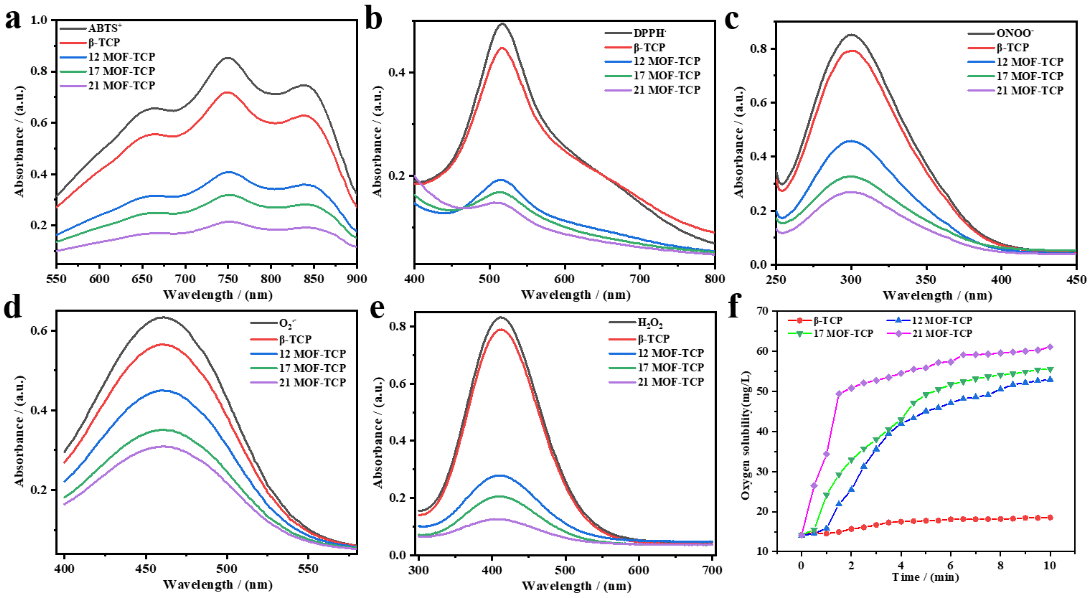

Figure S6. Multiple ROS-scavenging activities of MOF-TCP scaffolds. The absorbance spectra of (a) $ABTS^{+}$, (b) $DPPH^{\cdot}$, (c) $ONOO^{-}$, (d) $O_2^{\cdot -}$, and (e) H_2O_2 after treated with the β-TCP and MOF-TCP scaffolds. (f) O_2 produced from the H_2O_2 solution after treated with the β-TCP and MOF-TCP scaffolds.

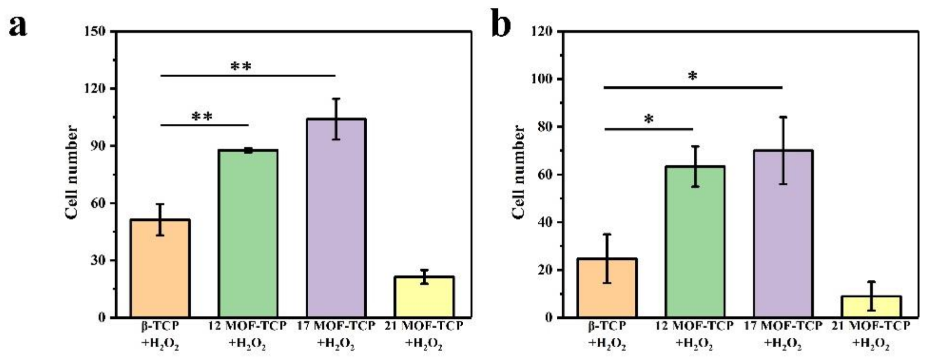

Figure S7. The number of cells that adhered to different scaffolds with H_2O_2 stimulation (n=3). *p < 0.05, **p < 0.01, ***p < 0.001.

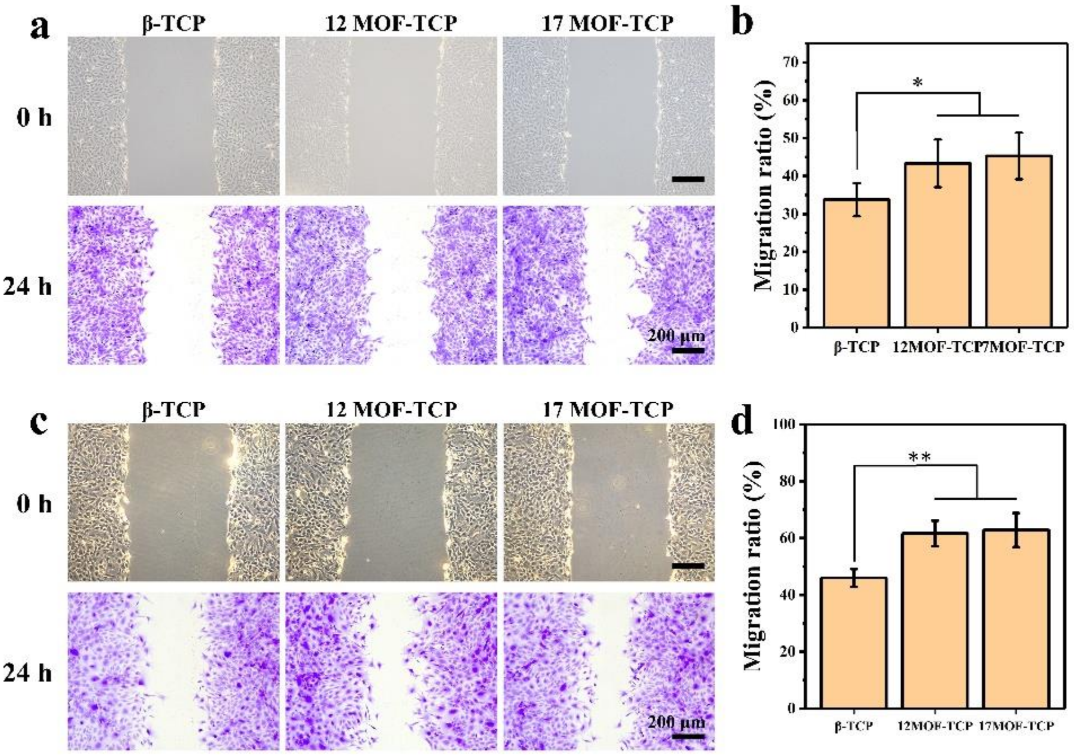

Figure S8. The promoting effects of MOF-TCP scaffolds on cell migration under oxidative stress. (a) The migration images of rBMSCs cultured with different scaffolds for 24 h under H_2O_2 stimulation. (b) Quantitative results of migration ratio of rBMSCs (n=4). (c) The migration images of chondrocytes cultured with different scaffolds for 24 h H_2O_2 stimulation. (d) Quantitative results of migration ratio of chondrocytes (n=4). $^*p < 0.05$, $^{**}p < 0.01$, $^{***}p < 0.001$.

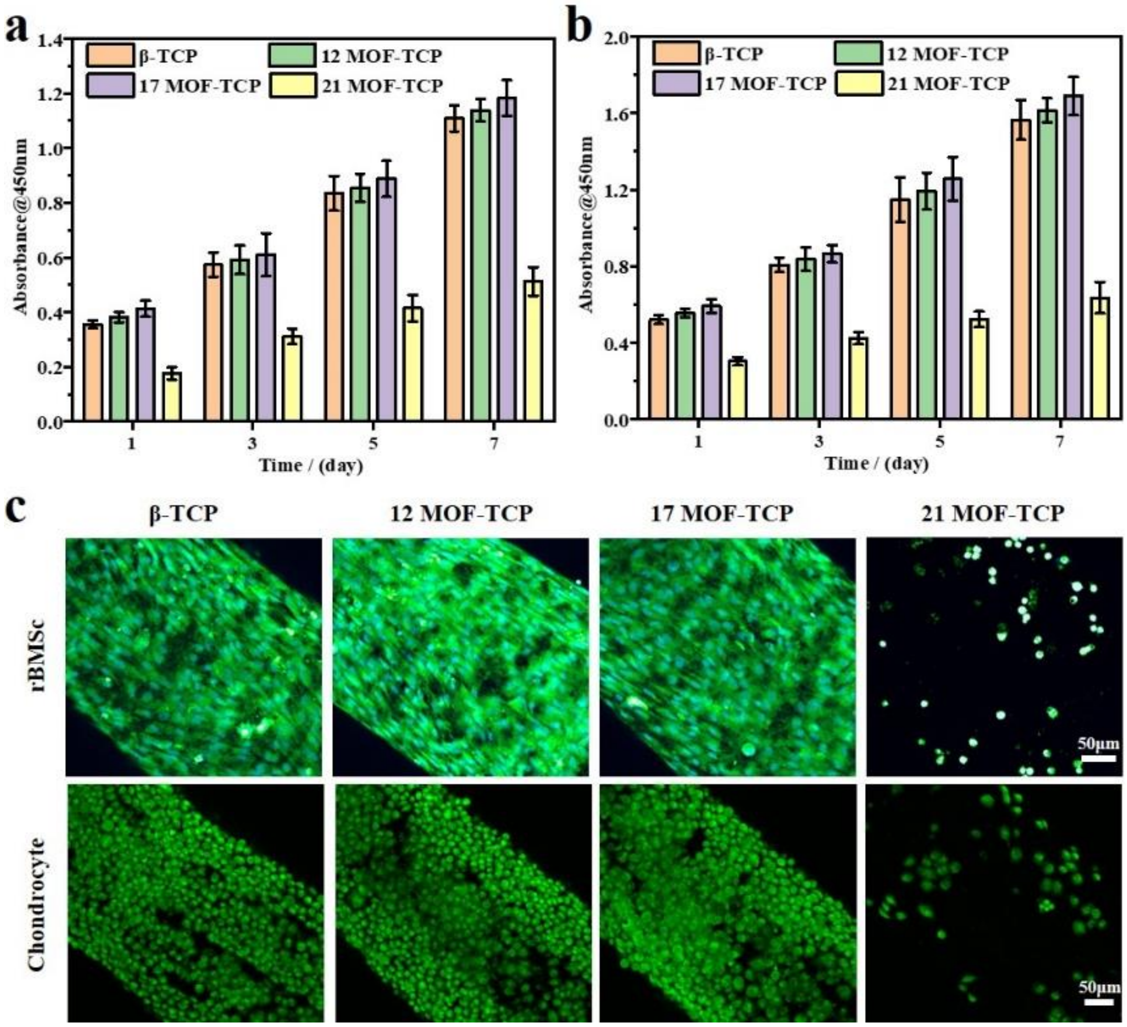

Figure S9. Proliferation of (a) rBMSCs and (b) Chondrocytes cultured on the scaffolds under normal conditions (n=6). (c) Morphology of rBMSCs and Chondrocytes cultured on the scaffolds under normal conditions.

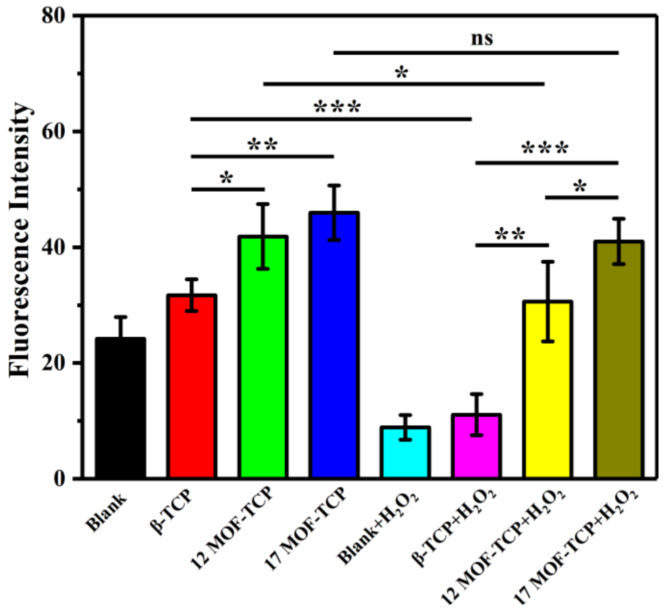

Figure S10. The semi-quantitative statistics of the fluorescence intensity of aggrecan protein in chondrocytes (n=4). *$p < 0.05$, **$p < 0.01$, ***$p < 0.001$.

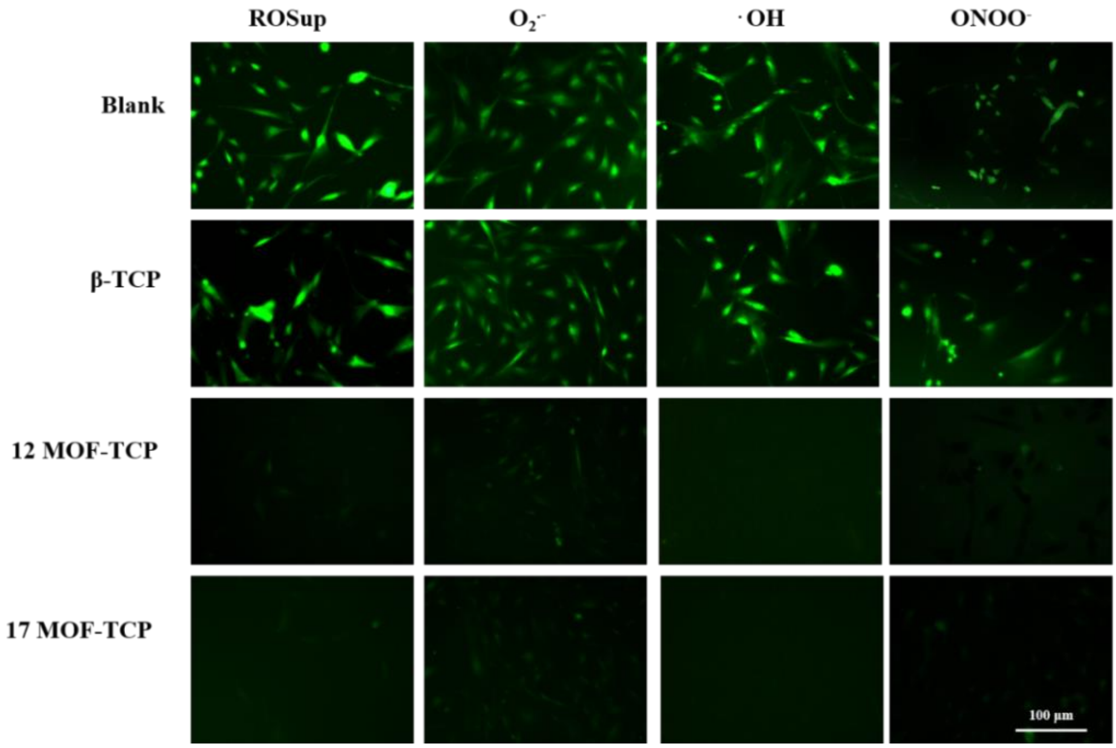

Figure S11. ROS fluorescence images in rBMSCs after being treated with different types of ROS and scaffolds.

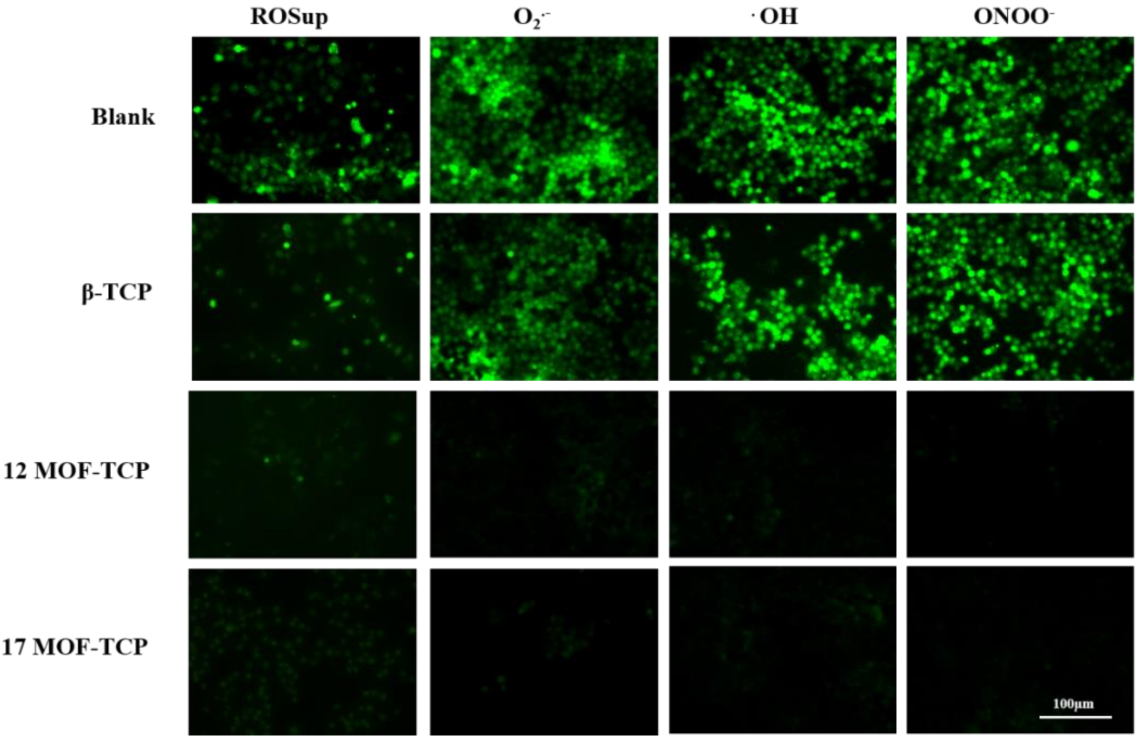

Figure S12. ROS fluorescence images in chondrocytes after being treated with different types of ROS and scaffolds.

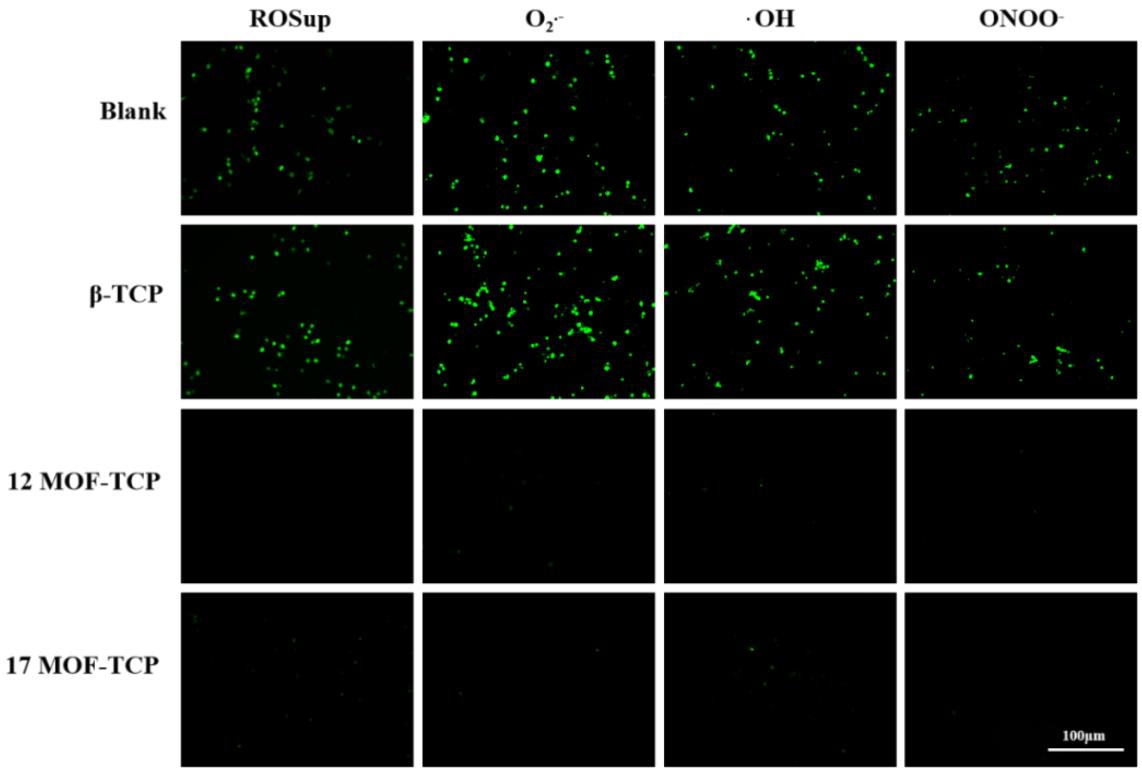

Figure S13. ROS fluorescence images in RAW 264.7 cells after being treated with different types of ROS and scaffolds.

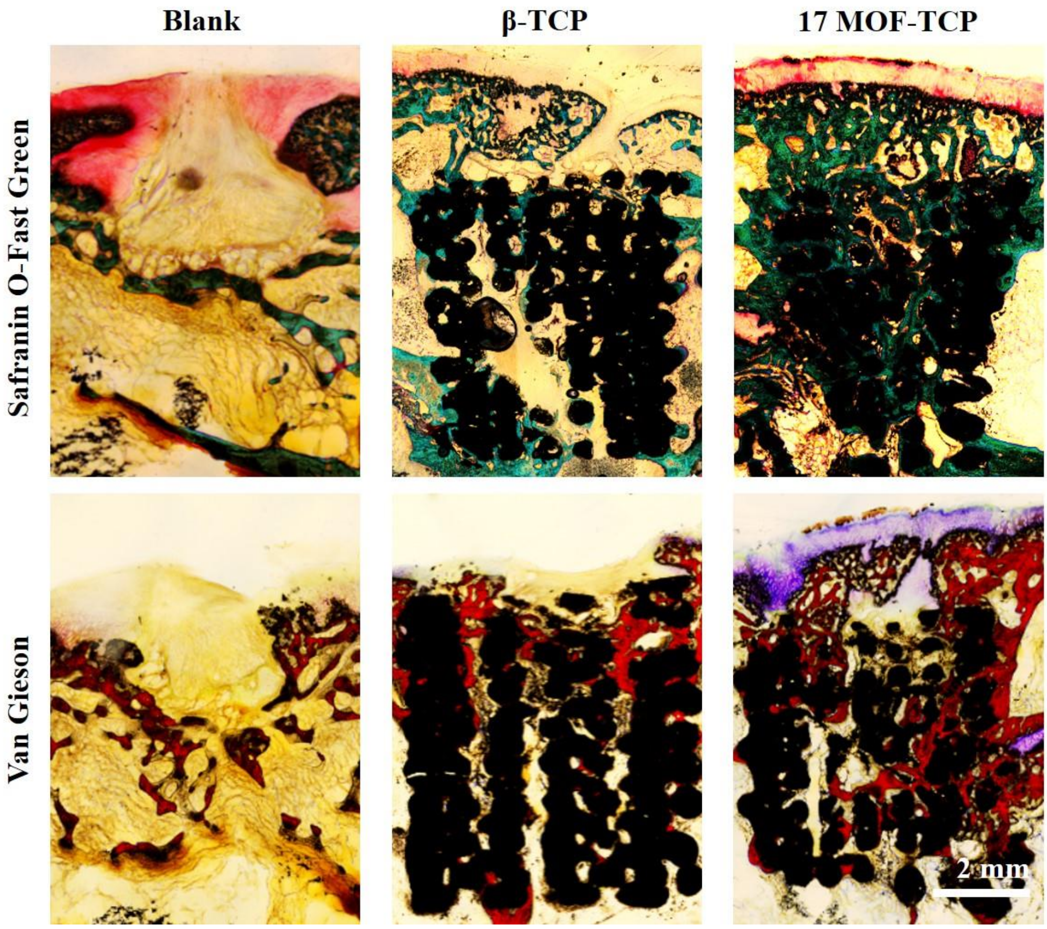

Figure S14. Safranine O-fast green staining images and Van Gieson staining images of the osteochondral defects after 12 weeks of implantation.

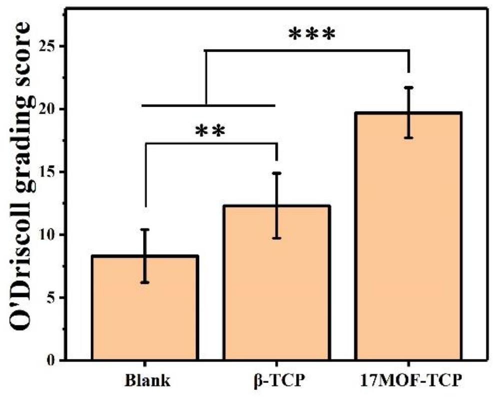

Figure S15. Quantitative analysis of the Safranine-O sections based on the O'Driscoll grading system (n=10). $^{**}p < 0.01$, $^{***}p < 0.001$.

Table S1. Proportions of Zn/Co-MOF reaction solutions for different MOF-TCP scaffolds

	A solution+H_2O/mL	B solution+H_2O/mL
6MOF-TCP	6.25+43.75	0.63+4.37
12MOF-TCP	12.50+37.50	1.25+3.75
17MOF-TCP	17.00+33.00	1.70+3.30
21MOF-TCP	21.00+29.00	2.10+2.90
25MOF-TCP	25.00+25.00	2.50+2.50
50MOF-TCP	50.00+0.00	5.00+0.00

Table S2. The primer sequences of osteogenic and chondrogenic genes used for RT-qPCR

Gene	Forward primer	Reverse primer
GAPDH	TCACCATCTTCCAGGAGCGA	CACAATGCCGAAGTGGTCGT
OCN	CCGGGAGCAGTGTGAGCTTA	AGGCGGTCTTCAAGCCATACT
OPN	CACCATGAGAATCGCCGT	CGTGACTTTGGGTTTCTACGC
BMP2	CGCCTCAAATCCAGCTGTAAG	GGGCCACAATCCAGTCGTT
RUNX2	TCAGGCATGTCCCTCGGTAT	TGGCAGGTAGGTATGGTAGTGG
SOX9	GGTGCTCAAGGGCTACGACT	GGGTGGTCTTTCTTGTGCTG
Aggrecan	AGGTCGTGGTGAAAGGTGTTG	GTAGGTTCTCACGCCAGGGA
COL-Ⅱ	AACACTGCCAACGTCCAGAT	CTGCAGCACGGTATAGGTGA

Table S3. The primer sequences of pro-inflammatory genes in chondrocytes used for RT-qPCR

Gene	Forward primer	Reverse primer
GAPDH	TCACCATCTTCCAGGAGCGA	CACAATGCCGAAGTGGTCGT
IL-1β	CAGGACCTGGACCTCTGCTGTC	GAGCCACAACGACTGACAAGACC
IL-6	GAAAACACCAGGGTCAGCAT	CAGCCACTGGTTTTTCTGCT
TNF-α	CTCCTACCCGAACAAGGTCA	CGGTCACCCTTCTCCAACT

Table S4. The primer sequences of anti-inflammatory and pro-inflammatory genes in macrophages used for RT-qPCR

GAPDH	AGAACATCATCCCTGCATCCAC	TCAGATCCACGACGGACACA
IL-10	GAGAAGCATGGCCCAGAAATC	GAGAAATCGATGACAGCGCC
Arg-1	AACCTTGGCTTGCTTCGGAACTC	GTTCTGTCTGCTTTGCTGTGATGC
IL-1β	CTACCTGTGTCTTTCCCGTG	TTTGTTGTTCATCTCGGAGC
IL-6	ATAGTCCTTCCTACCCCAATTTCC	GATGAATTGGATGGTCTTGGTCC
